# Systematic characterization of human gut microbiome-secreted molecules by integrated multi-omics

**DOI:** 10.1038/s43705-021-00078-0

**Published:** 2021-12-21

**Authors:** Bianca De Saedeleer, Antoine Malabirade, Javier Ramiro-Garcia, Janine Habier, Jean-Pierre Trezzi, Samantha L. Peters, Annegrät Daujeumont, Rashi Halder, Christian Jäger, Susheel Bhanu Busi, Patrick May, Wolfgang Oertel, Brit Mollenhauer, Cédric C. Laczny, Robert L. Hettich, Paul Wilmes

**Affiliations:** 1 Luxembourg Centre for Systems Biomedicine, University of Luxembourg, 7 Avenue des Hauts-Fourneaux, L-4362 Esch-sur-Alzette, Luxembourg; 2 Integrated Biobank of Luxembourg, Luxembourg Institute of Health, 1, rue Louis Rech, L-3555 Dudelange, Luxembourg; 3 Oak Ridge National Laboratory, 1 Bethel Valley Road, Oak Ridge, TN 37830, USA; 4 Department of Neurology, Philipps-University Marburg, Baldinger Str. 1, 35043 Marburg, Germany; 5 Department of Neurology, University Medical Center Goettingen, Robert-Koch-Str. 40, 37075 Goettingen, Germany; 6 Paracelsus-Elena-Klinik, Klinikstr. 16, 34128 Kassel, Germany; 7 Department of Life Sciences and Medicine, Faculty of Science, Technology and Medicine, University of Luxembourg, 6 Avenue du Swing, L-4367 Belvaux, Luxembourg

## Abstract

The human gut microbiome produces a complex mixture of biomolecules that interact with human physiology and play essential roles in health and disease. Crosstalk between micro-organisms and host cells is enabled by different direct contacts, but also by the export of molecules through secretion systems and extracellular vesicles. The resulting molecular network, comprised of various biomolecular moieties, has so far eluded systematic study. Here we present a methodological framework, optimized for the extraction of the microbiome-derived, extracellular biomolecular complement, including nucleic acids, (poly)peptides, and metabolites, from flash-frozen stool samples of healthy human individuals. Our method allows simultaneous isolation of individual biomolecular fractions from the same original stool sample, followed by specialized omic analyses. The resulting multi-omics data enable coherent data integration for the systematic characterization of this molecular complex. Our results demonstrate the distinctiveness of the different extracellular biomolecular fractions, both in terms of their taxonomic and functional composition. This highlights the challenge of inferring the extracellular biomolecular complement of the gut microbiome based on single-omic data. The developed methodological framework provides the foundation for systematically investigating mechanistic links between microbiome-secreted molecules, including those that are typically vesicle-associated, and their impact on host physiology in health and disease.

High-throughput sequencing and its applications have produced new insights into the human gut microbiome’s structural diversity [1] and functional potential [2]. In health and disease, the gut microbiome confers essential functionalities [3] by interfacing directly with human metabolism [4] as well as ensuring intestinal homeostasis and immune system stimulation [3], among others [2]. Microbiome-secreted molecules, including nucleic acids, (poly) peptides, enzymes, and metabolites, play key roles in microbiomehost signaling [5] and are released into the human gastrointestinal tract via secretory systems and/or outer membrane vesicles (OMVs) [5]. Substantial differences exist between predicted functionalities based on metagenomic analyses and actual microbial phenotypes in the gut [2]. The immunogenic potential of commensals and pathobionts thereby remains largely unexplored, especially as the emergent properties of the microbiome in relation to host interactions remain to be comprehensively characterized and understood. Moreover, the fraction of genes encoding proteins of unknown function constitutes between 40 and 70% of genes, and such proteins constitute half of those that are identifiable in metaproteomic data from fecal protein extracts [2]. Further exacerbating the situation concerning such unknowns is the fact that the majority of gut microbiome-derived small molecules (>90%) do not have any references in public databases despite their immediate relevance to host physiology [6]. Finally, RNA transcripts reflect microbial viability and affect antibody responses [7] but microbiome-derived extracellular small and large RNAs in the gastrointestinal tract remain largely uncharacterized [8]. Collectively, the diversity of microbiome-secreted biomolecules involved in host-microbiome interactions is vast and comprises an extensive array of so far unexplored material.

To obtain an overview of this diversity, we developed a framework to systematically characterize the extracellular complement of microbiome-derived molecules including DNA (ex-DNA), small and large RNA (ex-sRNA and ex-lRNA), (poly)peptides (ex-Prot), and metabolites [polar metabolites, short-chain fatty acids (SCFAs), and bile acids (BAs)] from the human gut by integrated multi-omics (Supplementary Materials and Methods). The present work thereby represents a systematic and extensive expansion of the previous methodological workflow by Roume et al. [9], which focused on the intracellular biomolecular complements. Moreover, we analyze and contextualize the resulting extracellular high-resolution multi-omics data. Briefly, using our new method, snap-frozen stool samples from four healthy individuals are homogenized and are subjected to an optimized biomolecular isolation method [9] (Fig. 1A). Isolation and purification of the intracellular molecules are performed after cell lysis on the resuspended pellet using silica-column-based techniques. For the extracellular fractions, fecal water is recovered using low-speed centrifugation and low-flow filtration to avoid microbial cell lysis [9]. All obtained nucleic acid fractions are subjected to high-throughput sequencing. Peptides are isolated after precipitation using trichloroacetic acid and sodium deoxycholate to ensure recovery of low abundance (poly)peptides. Ex-Prot are subjected to SDS-PAGE electrophoresis followed by LC with tandem mass spectrometry (LC-MS/MS). Metabolites are extracted by adding the respective internal standards, followed by recovery of the phase of interest. Metabolite fractions are analyzed using combinations of gas chromatography-mass spectrometry (GC-MS) and liquid chromatography coupled to high-resolution mass spectrometry (LC-HRMS). To allow integrated taxonomic and functional analyses, reference metagenome assembled genomes (MAGs), against which the extracellular nucleic acid fractions are mapped and which are used for protein identifications, are obtained by coassembling the intracellular nucleic acid data using the Integrated Meta-omics Pipeline (IMP) [10]. Subsequently, based on the resulting genomic foundation, the metaproteomic data are further integrated via matching of the mass spectra using the contig-derived databases for protein identification. In addition, the identified metabolites may be integrated via their annotation to reactions and their corresponding enzymes derived from the above integrated analyses. An example of an integrated analysis view is shown in Supplementary Figs. S1 and S2.

The individual extracellular complements were effectively extracted using our methodology (Fig. 1B, Supplementary Figs. S3–S5, and Supplementary Tables S1–S3). Interestingly, proteins were over-represented and nucleic acids underrepresented when compared to the average intracellular composition of a bacterium [11]. We compared the intracellular composition of Escherichia coli as defined by Neidhardt et al. [11] to the extracellular fractions we obtained (Supplementary Fig. S6). Our observations, including the overrepresentation of proteins in the extracellular fractions, are expected as most of the macromolecular export machinery within a microbial cell is selective for protein export. An example for this being all proteins tagged with signal peptides and those exported via bacterial secretion systems such as Sec, Tat, Type-1 to Type-9 secretion systems [12]. On the other hand, nucleic acid export is known to occur primarily via conjugation or transduction and occurs between cells rather than the extracellular compartment. The exception to this is the export of nucleic acids via extracellular vesicles (EVs). Our protocol is also designed to capture the EVs in the extracellular fraction, whereby the centrifugation speed is set up to separate cells from the entire extracellular content. Taxonomic assignment based on the MAGs as well as the functional annotations demonstrated the uniqueness of the different biomolecular fractions whereby the int-DNA, as solely used for a typical metagenomic analysis, did not allow inferences regarding the composition of the extracellular complements (Fig. 2A). For example, dominant gut microbiome taxa and organisms of interest, e.g., Roseburia spp., were differentially represented in the different fractions (Supplementary Fig. S7). We also found that Blautia spp. was significantly differentially represented between the various fractions (Supplementary Table S4). In addition, the overall taxonomic composition showed higher variation between fractions and individuals than the corresponding functional representations (Fig. 2, Supplementary Fig. S8, and Supplementary Table S5 and S6). We also observed differences at the functional levels between the int-DNA and other fractions with respect to genes encoding for tRNAs and other functions (Supplementary Table S7). Since int-DNA is solely used in typical metagenomic studies, we assessed the overlap between int-DNA and the other extracellular fractions. The differences were apparent in the overlap between the assessed fractions at the nucleotide (Supplementary Fig. S9), taxonomic (Supplementary Fig. S10), and functional levels (Supplementary Fig. S11), thereby underlining the necessity for the systematic characterization of the individual fractions. Importantly, the resolved inter- and intraindividual variations are in line with our previous work focused on the intracellular fractions [2], thereby reinforcing the notion that the individual is the largest contributor to the observed variation within the microbiome-derived biomolecular fractions.

With respect to host-microbiome interactions especially in relation to immunostimulation, the ex-DNA along with the ex-lRNA contained genes from pathobionts, e.g., Staphylococcus spp., known to alter IL-8 expression via recognition of CpG motifs by TLR9 [13]. The ex-lRNA fraction was enriched in RNAs derived from specific bacterial taxa, e.g., Faecalibacterium spp. (comprising up to 22% of reads; Fig. 2A and Supplementary Table S8), and RNA viruses, e.g., tobacco mosaic virus (up to 8%). Furthermore, we observed a general enrichment in non-coding RNAs (ncRNAs; up to 57%; Fig. 2B and Supplementary Table S9). Interestingly, human gut-associated archaea such as Methanobrevibacter smithii represented up to 5% in Individual 3 (Supplementary Fig. S12 and Supplementary Table S8). M. smithii’s RNA is known to trigger TLR8-dependent NLRP3 inflammasome activation [14]. The ex-sRNA fractions were enriched in sequences from different members of the Clostridiales (up to 43%; Fig. 2A and Supplementary Table S8), mainly being transfer-RNAs (tRNAs; 91–97%), ribosomal RNAs (rRNAs; 0.2–3%), or other non-coding RNAs (ncRNAs; 1–4%; Fig. 2B and Supplementary Table S9).

We captured specific molecules that are typically enriched in bacterial OMVs including several 50S ribosomal proteins encoded by the *rplE*, *rplL*, *rplM*, and *rplY* genes [15], mainly originating from the Bacteroidales (Supplementary Table S10). Overall, the nucleic acid fractions contained genes coding for various vesicle-associated proteins that were also present among the ex-Prot. Examples include chaperone protein HtpG [15] and the outer membrane proteins OmpA, OmpF, FepA, and BamA [16] (Supplementary Table S11). The majority were derived from Bacteroidales and Gammaproteobacteria (Supplementary Table S10). Furthermore, we detected multiple enzymes, known to be enriched in OMVs, such as, glutamine synthetase (glnA), protein recombinase A (*recA*) [14], and formate acetyltransferase 1 (*pflB*) [16] (Supplementary Table S11). These were specifically encoded by different members of the Bacteroidales (Supplementary Table S10). This indicates the ability of our newly developed protocol to resolve vesicle-associated biomolecules along with soluble molecules. The functional repertoires of the ex-Prot exhibited mainly involvement in transport and metabolism of components (60–63%; Fig. 2B and Supplementary Table S9), thereby indicating distinct export mechanisms and specific enrichments in the extracellular space.

The metabolome contained microbiota-secreted molecules such as SCFAs, secondary BAs (Fig. 2C and Supplementary Table S12), and derivatives (Supplementary Fig. S13 and Supplementary Table S13), known to play crucial roles in host metabolism, immune, and inflammatory pathways [4]. For example, lithocholic acid derivatives inhibit T_h_17 cell differentiation and stimulate Treg differentiation [17]. Furthermore, formate provides a substrate for Enterobacteriaceae expansion in the gut, which intensifies inflammation-associated dysbiosis [18]. Acetate, butyrate, and propionate contribute to the anti/pro-inflammatory equilibrium, their imbalance has been linked to chronic inflammation eventually leading to various autoimmune diseases [19].

It is challenging to distinguish host- versus gut microbiome-derived biomolecules, especially for those that cannot be immediately linked back to the genomic information such as is the case for metabolites. For instance, with respect to DNA, host DNA can be identified in silico during the assembly step (see Methods), allowing the distinction between bacterial and host- derived DNA. Aside from this, mammalian mRNA may be distinguished from microbial transcripts based on the presence of a polyA tail in the former. The exceptions here, however, include commensal eukaryotes such as fungi and Blastocystis, some sRNAs, and non-polyadenylated molecules [20]. For the majority of the proteins, based on the genomic foundation, we have previously described that systematic omic measurements in a tight coupling with experimental approaches allow for the inference of causal relationships via coherent data integration [2, 21]. This approach, in addition to organismal affiliation of metabolites, may be fruitful in the context of organismal assignments of non-ribosomal peptides. Furthermore, in the context of metabolites, a top-down approach has recently been demonstrated by Zimmerman et al. [22], whereby specific microbiota-derived metabolites, especially in the context of drug metabolism, were differentiated from those of the host. More broadly speaking, metabolites may also be attributable to organisms via metabolic reconstructions, either at the communitylevel [23, 24] or taxon-level [25], in a complementary bottom-up approach. In the context of molecule-to-organism linkages, the generation of systematic high-resolution data along with appropriate data analytical methods can establish relevant associations, which then need to be further validated experimentally [2]. In this context, our expanded biomolecular isolation methodology presented here provides the foundation for identifying such relationships following precise and multi-dimensional analyses from the same original sample that is critical for coherent multi-omics data integration [26]. This is particularly relevant when working on heterogenous microbiome samples such as stool. We note that our herein described biomolecular extraction methodology should be generally applicable to other sample types such as saliva, skin, or vaginal samples. The main limitation in this context is associated with the yield of the extractions, i.e., the mentioned sample types yield lower cell numbers compared to fecal samples. If this bottleneck is carefully considered and related adjustments are made, our method, as it is based inter alia on indiscriminate cryogenic lysis of cells [9], should be generally applicable to extract from other sample types and subsequently perform meaningful omic measurements. Several chronic diseases are thought to have a constitutively (pro)-inflammatory state, potentially underlying disease etiology [27]. Therefore, given the distinctiveness of the extracellular biomolecular fractions and their involvement in modulating immune and inflammatory pathways, deciphering this molecular complex and its effect on the human host represents one of the many challenges to be faced in the coming years. Thereby, our results support the notion that the integration of additional omics data beyond metagenomics (based on int-DNA) adds essential dimensions in terms of taxonomic and functional information, not least in relation to likely effector biomolecules. Our methodology thereby represents the foundation for the systematic study of the gut microbiome’s extracellular molecular complex in the context of human health and disease.

## Supplementary Material

Supplementary Materials and Methods

Supplementary Table S1

Supplementary Table S2

Supplementary Table S3

Supplementary Table S4

Supplementary Table S5

Supplementary Table S6

Supplementary Table S7

Supplementary Table S8

Supplementary Table S9

Supplementary Table S10

Supplementary Table S11

Supplementary Table S12

Supplementary Table S13

## Figures and Tables

**Fig. 1:**
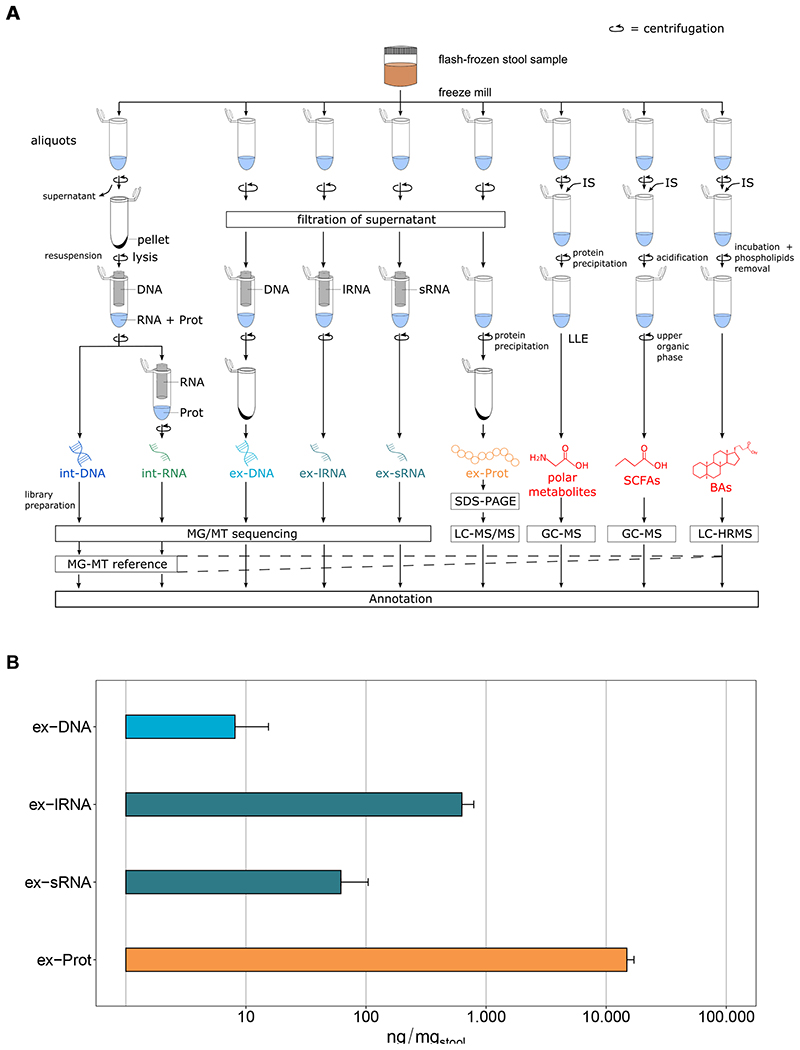
Overview of the methodological workflow and characteristics of the obtained biomolecular fractions. **A** Flowchart of the experimental and bioinformatic analyses. Flash-frozen stool samples are divided into aliquots for subsequent biomolecular extractions. Int-DNA are obtained after elution of the lysate bound onto an AllPrep DNA spin column, the flow-through is loaded onto a RNeasy spin column for int-RNA isolation. To obtain the extracellular fractions, the supernatant is first filtered through a polyethersulfone (PES) membrane. Nucleic acid fractions are isolated using specific columns (NucleoSpin miRNA Plasma kit for ex-DNA and ex-sRNA, NucleoSpin RNA Blood kit for ex-lRNA). Ex-DNAs are subjected to an additional concentration step. All nucleic acid fractions are subjected to high-throughput sequencing. ExProt are obtained from the resulting pellet after protein precipitation and analyzed by SDS-PAGE followed by LC-MS/MS. The sequencing information from the intracellular fractions allows for genome reconstruction by a DNA-RNA co-assembly using IMP [10]. This MG-MT reference allows further mapping and annotation of the extracellular fractions. Polar metabolites, SCFAs, and BAs are extracted from their respective aliquots by addition of specific internal standards (IS) and further processing of the supernatant (Supplementary Materials and Methods). The extracts are then analyzed by GC-MS, GC-MS, and LC-HRMS, respectively. **B** Masses of biomolecules extracted per mg of original stool sample (logarithmic scale). Error bars represent standard deviation on four independent samples. ex-DNA extracellular DNA, ex-sRNA extracellular small RNA, ex-lRNA extracellular large RNA, ex-Prot extracellular proteins, SCFAs short-chain fatty acids, BAs bile acids.

**Fig. 2:**
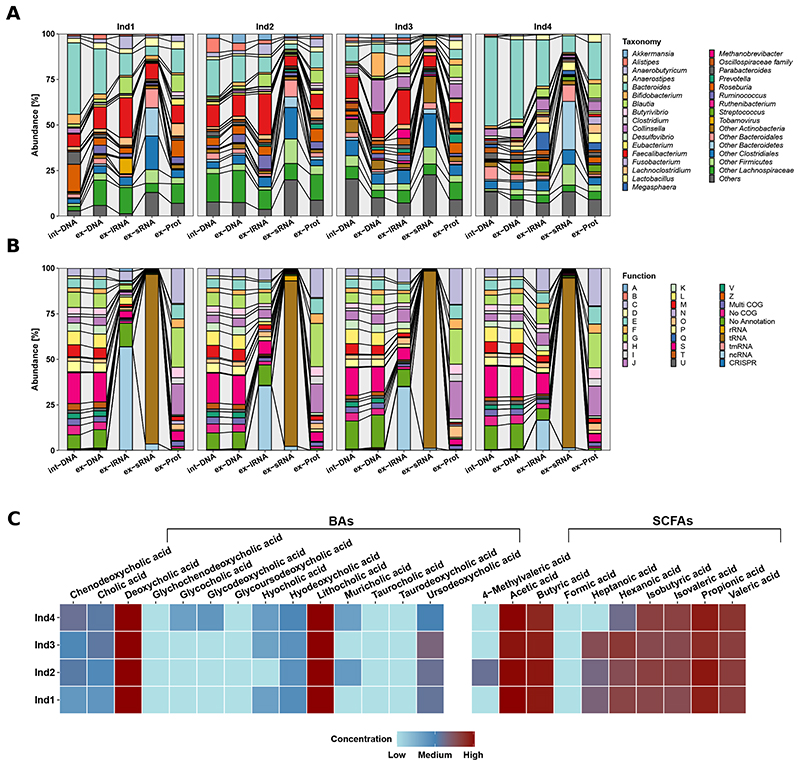
Composition of the extracted biomolecular fractions from gut microbiome samples of four healthy human individuals. **A** Relative abundance (%) of the taxonomic annotations at the genus level based on the co-assembled contigs using Kraken2. Differences in composition are observed between the different fractions as well as between the individuals (Ind). **B** Relative abundance (%) of the functional classification on the co-assembled contigs according to clusters of orthologous groups (COGs) and non-coding RNA types. Abbreviations of the functional categories: A: RNA processing and modification; B: chromatin structure and dynamics; C: energy production and conversion; D: cell cycle control: cell division: chromosome partitioning; E: amino acid transport and metabolism; F: nucleotide transport and metabolism; G: carbohydrate transport and metabolism; H: coenzyme transport and metabolism; I: lipid transport and metabolism; J: translation: ribosomal structure and biogenesis; K: transcription; L: replication: recombination and repair; M: cell wall/membrane/envelope biogenesis; N: cell motility; O: post-translational modification: protein turnover and chaperones; P: inorganic ion transport and metabolism; Q: secondary metabolites biosynthesis: transport and catabolism; S: function unknown; T: signal transduction mechanisms; U: intracellular trafficking: secretion and vesicular transport; V: defense mechanisms; Z: cytoskeleton. **C** Heatmap of the bile acid (BA) and short-chain fatty acid (SCFA) concentrations (μg/L; logarithmic scale), measured by GC-MS and LC-HRMS, respectively, for each individual. Lower concentrations are indicated in blue and range from 0 to 98,029.2 μg/L, higher concentrations are shown in red, ranging from 98,029.2 to 196,058.4 μg/L. SCFAs are originally measured in μmol/L in a dynamic range from 10 to 4000 μmol/L, BAs are measured in ng/mL ranging from 50 to 4000 ng/mL. BAs bile acids, SCFAs short-chain fatty acids, int-DNA intracellular DNA, ex-DNA extracellular DNA, ex-sRNA extracellular small RNA, ex-lRNA extracellular large RNA, ex-Prot extracellular proteins.

## Data Availability

The raw sequence libraries are deposited in the European Nucleotide Archive (ENA) at EMBL-EBI under accession number PRJEB44766. The raw MS files are deposited in the MassIVE, ProteomeXchange, and PRIDE databases under the experiment accession numbers MSV000086973 and PXD024472, respectively. Supplementary Tables S8, S10, and S11 are available on Figshare (https://doi.org/10.6084/m9.figshare.c.5694595.v1).

## References

[1] Segata N, Boernigen D, Tickle TL, Morgan XC, Garrett WS, Huttenhower C (2013). Computational meta’omics for microbial community studies. Mol Syst Biol.

[2] Heintz-Buschart A, Wilmes P (2018). Human gut microbiome: function matters. Trends Microbiol.

[3] Hooper LV, Littman DR, Macpherson AJ (2012). Interactions between the microbiota and the immune system. Science.

[4] Sonnenburg JL, Backhed F (2016). Diet-microbiota interactions as moderators of human metabolism. Nature.

[5] Ghosal A (2017). Importance of secreted bacterial RNA in bacterial-host interactions in the gut. Microb Pathog.

[6] Peisl BYL, Schymanski EL, Wilmes P (2018). Dark matter in host-microbiome metabo-lomics: tackling the unknowns–a review. Anal Chim Acta.

[7] Barbet G, Sander LE, Geswell M, Leonardi I, Cerutti A, Iliev I (2018). Sensing microbial viability through bacterial RNA augments T follicular helper cell and antibody responses. Immunity.

[8] Fritz JV, Heintz-Buschart A, Ghosal A, Wampach L, Etheridge A, Galas D (2016). Sources and functions of extracellular small RNAs in human circulation. Annu Rev Nutr.

[9] Roume H, Muller EL, Cordes T, Renaut J, Hiller K, Wilmes P (2013). A biomolecular isolation framework for eco-systems biology. ISME J.

[10] Narayanasamy S, Jarosz Y, Muller EEL, Heintz-Buschart A, Herold M, Kaysen A (2016). IMP: a pipeline for reproducible reference-independent integrated meta-genomic and metatranscriptomic analyses. Genome Biol.

[11] Neidhardt FC, Neidhardt N, Frederick C, Ingraham JL, Schaechter M (1990). Physiology of the bacterial cell: a molecular approach. Sinauer Associates; Biochemical Education.

[12] Guerrero-Mandujano A, Hernández-Cortez C, Ibarra JA, Castro-Escarpulli G (2017). The outer membrane vesicles: secretion system type zero Traffic.

[13] Dalpke A, Frank J, Peter M, Heeg K (2006). Activation of toll-like receptor 9 by DNA from different bacterial species. Infect Immun.

[14] Vierbuchen T, Bang C, Rosigkeit H, Schmitz RA, Heine H (2017). The human-associated archaeon methanosphaera stadtmanae is recognized through its RNA and induces TLR8-dependent NLRP3 inflammasome activation. Front Immunol.

[15] Taheri N, Mahmud AKMF, Sandblad L, Fällman M, Wai SN, Fahlgren A (2018). Campylobacter jejuni bile exposure influences outer membrane vesicles protein content and bacterial interaction with epithelial cells. Sci Rep.

[16] Hong J, Dauros-Singorenko P, Whitcombe A, Payne L, Blenkiron C, Phillips A (2019). Analysis of the Escherichia coli extracellular vesicle proteome identifies markers of purity and culture conditions. J Extracell Vesicles.

[17] Hang S, Paik D, Yao L, Kim E, Trinath J, Lu J (2019). Bile acid metabolites control T H 17 and T reg cell differentiation. Nature.

[18] Hughes ER, Winter MG, Duerkop BA, Spiga L, Furtado de Carvalho T, Zhu W (2017). Microbial respiration and formate oxidation as metabolic signatures of inflammation-associated dysbiosis. Cell Host Microbe.

[19] Martin CR, Osadchiy V, Kalani A, Mayer EA (2018). The brain-gut-microbiome axis. Cell Mol Gastroenterol Hepatol.

[20] Yang L, Duff MO, Graveley BR, Carmichael GG, Chen LL (2011). Genomewide characterization of non-polyadenylated RNAs. Genome Biol.

[21] Muller EE, Glaab E, May P, Vlassis N, Wilmes P (2013). Condensing the omics fog of microbial communities. Trends Microbiol.

[22] Zimmermann M, Zimmermann-Kogadeeva M, Wegmann R, Goodman AL (2019). Mapping human microbiome drug metabolism by gut bacteria and their genes. Nature.

[23] Greenblum S, Turnbaugh PJ, Borenstein E (2012). Metagenomic systems biology of the human gut microbiome reveals topological shifts associated with obesity and inflammatory bowel disease. Proc Natl Acad Sci USA.

[24] Roume H, Heintz-Buschart A, Muller EEL, May P, Satagopam VP, Laczny CL (2015). Comparative integrated omics: identification of key functionalities in microbial community-wide metabolic networks. NPJ Biofilms Microbiomes.

[25] Magnúsdóttir S, Heinken A, Kutt L, Ravcheev DA, Bauer E, Noronha A (2017). Generation of genome-scale metabolic reconstructions for 773 members of the human gut microbiota. Nat Biotechnol.

[26] Heintz-Buschart A, May P, Laczny CC, Lebrun LA, Bellora C, Krishna A (2017). Integrated multi-omics of the human gut microbiome in a case study of familial type 1 diabetes. Nat Microbiol.

[27] Furman D, Campisi J, Verdin E, Carrera-Bastos P, Targ S, Franceschi C (2019). Chronic inflammation in the etiology of disease across the life span. Nat Med.

