# Supplementary material for: Systematic characterization of human gut microbiome-secreted molecules by integrated multi-omics
Source: ISME Commun. 2021 Dec 21;1:82. doi: 10.1038/s43705-021-00078-0 (PMC7612290; doi:10.1038/s43705-021-00078-0)
Supplement: Supplementary file 1 — Supplementary Methods and Figures [file 43705_2021_78_MOESM1_ESM.pdf]

Supplementary information

Systematic characterization of human gut microbiome-secreted molecules by integrated multi-omics

Bianca De Saedeleer[1], Antoine Malabirade[1], Javier Ramiro-Garcia[1], Janine Habier[1], Jean-Pierre Trezzi[1,2], Samantha L. Peters[3], Annegrät Daujeumont[1], Rashi Halder[1], Christian Jäger[1], Susheel Bhanu Busi[1], Patrick May[1], Wolfgang Oertel[4], Brit Mollenhauer[5,6], Cédric C. Laczny[1], Robert L. Hettich[3] and Paul Wilmes[1,7*]

[1] Luxembourg Centre for Systems Biomedicine, University of Luxembourg, 7 avenue des Hauts-Fourneaux L-4362 Esch-sur-Alzette, Luxembourg.

[2] Integrated Biobank of Luxembourg, Luxembourg Institute of Health, 1, rue Louis Rech, L-3555 Dudelange, Luxembourg.

[3] Oak Ridge National Laboratory, 1 Bethel Valley Road, Oak Ridge, Tennessee, 37830, USA.

[4]Department of Neurology, Philipps-University Marburg, Baldinger Str. 1, 35043 Marburg, Germany

[5]Department of Neurology, University Medical Center Goettingen, Robert-Koch-Str. 40, 37075 Goettingen

[6] Paracelsus-Elena-Klinik, Klinikstr. 16, 34128 Kassel

[7]Department of Life Sciences and Medicine, Faculty of Science, Technology and Medicine, University of Luxembourg, 6 avenue du Swing, L-4367 Belvaux, Luxembourg.

*Correspondence: paul.wilmes@uni.lu

This document includes:

Supplementary Materials and Methods

Supplementary References

Supplementary Note

Supplementary Figures S1 to S13

Supplementary Tables S1 to S13

Supplementary Materials and Methods

Sample collection and handling

Stool samples were collected from four healthy human volunteers. Samples were immediately flash-frozen in liquid nitrogen and stored at -80°C to guarantee optimal sample integrity and quality. Sample taking conformed to the Declaration of Helsinki and was approved by the ethics committee of the Physician's Board Hessen, Germany (FF 38/2016). The collected frozen stool samples were homogenized (6875D Freezer/Mill Spex - Instrument Solutions Benelux BV) and separated into 150 mg aliquots for subsequent biomolecular extractions. Each individual sample was measured to allow relative corrections of the fecal mass quantifications. Samples for bio-macromolecular extractions were incubated in RNAlater-ice, at -20°C, overnight prior to RNA/DNA/Protein extractions.

Biomolecular extractions

A summarized overview of the extraction protocol is depicted in Figure 1A.

Intracellular nucleic acids

Three cold autoclaved 4 mm stainless steel milling balls (Retsch) were added to each 150 mg stool aliquot which were then homogenized by shaking for 2 min at 10 Hz in an Oscillating Mill MM 400 (Retsch), followed by centrifugation at 700 g for 2 min at 4°C. The supernatant was collected into a 2 mL Eppendorf tube and was subsequently centrifuged at 14 000 g for 5 min at 4°C. After removal of the supernatant, cooled (by use of liquid nitrogen) stainless steel milling balls (5x(2mm) + 2x(5mm)]) together with 600 µl of cold RLT buffer (containing 10 µl/ml of 2-mercaptoethanol) were added to the resulting interphase pellet. The tubes were then covered

with parafilm prior to re-suspension of the pellet by quick vortexing. Bead beating was performed for 30 seconds at 25 Hz in cold racks (4°C; Oscillating Mill MM 400, Retsch®). The resulting lysate was loaded onto a QIAshredder column and centrifuged for 2 min at 12 000 g. The flow-through was then transferred to an AllPrep DNA spin column (Qiagen) placed in a 2 ml collection tube and centrifuged for 1 min at 12 000 g. The AllPrep DNA spin column was placed in a new 2 mL collection tube (supplied) and stored at 4°C for later DNA purification.

The flow-through was used for the RNA purification using the AllPrep RNA Mini kit (Qiagen). First, 400 µl of pure ethanol were added to the flow-through and mixed by pipetting. For each sample, 700 µl were loaded onto an RNeasy spin column, which was placed in a 2 ml collection tube (supplied), and centrifuged for 1 min at 12 000 g. The flow-through was discarded. The spin column membrane was washed by first adding 700 µl Buffer RW1 and centrifuging for 1 min at 12 000 g. Then, 500 µl Buffer RPE were added to the RNeasy spin column and centrifuged for 1 min at 12 000 g. To wash the spin column membrane, 500 µl Buffer RPE were added to the RNeasy spin column again and centrifuged for 2 min at 12 000 g. The long centrifugation dries the spin column membrane, ensuring that no ethanol is carried over during RNA elution, as residual ethanol may interfere with downstream reactions. After centrifugation, the RNeasy spin column was carefully removed from the collection tube, avoiding contacting the flow-through. Otherwise, carryover of ethanol would have occurred. The collection tube containing the flow-through was discarded. To eliminate any possible carryover of Buffer RPE, or if residual flow-through remains on the outside of the RNeasy spin column, the column was placed in a new 2 ml collection tube and centrifuged for 1 min at 12 000 g. To elute the RNA, the RNeasy spin column was placed in a new 1.5 ml collection tube, 50 µl RNase-free water was added directly to the spin column membrane and centrifuged for 1 min at 12 000 g. This elution step was repeated once in a new tube.

Purification of the genomic DNA was obtained by use of the AllPrep DNA Mini kit (Qiagen). The AllPrep DNA spin column was first washed by adding 500 µl Buffer AW1 and centrifugation for 1 min at 12 000 g. This step was repeated with 500 µl Buffer AW2 and centrifugation for 2 min. After placing the spin column in a new 1.5 ml collection tube, 100 µl Buffer EB (preheated to 70°C) were directly added to the spin column membrane and incubated at room temperature (15–25°C) for 2 min, and then centrifuged for 1 min at 12 000 g to elute the DNA. This elution step was repeated once in a new tube.

Extracellular extraction of nucleic acids

150 mg stool aliquots were gently resuspended in 900 µl of ice-cold sterile 1X D-PBS buffer (Sigma) to recover faecal water and any other extracellular material. In the case of RNA isolation, 1 µl of Riboguard RNase Inhibitor at 100 U/µl was added (Lucigen). The resuspended samples were centrifuged at 10 000 g at 4°C for 10 min and the recovered supernatant was filtered through a 0.2 µm PS membrane. As the quantity in material from the extracellular space was expected to be lower than within cells, extracellular nucleic acid isolation was performed using cell-free extraction kits. These are optimized for extracting low amounts of material from circulating fluids. For each isolated fraction, a blank extraction was also included and subjected to an identical protocol to check for the possible presence of any external contaminations (Table S3). Small RNA was purified using the NucleoSpin miRNA Plasma kit (Macherey-Nagel) according to the manufacturer's instructions, including on-column rDNase digestion to remove contaminant DNA. RNA was eluted in 30 µl nuclease-free water. DNA was purified using the same kit, omitting rDNase treatment. DNA was eluted in 100 µl of ultrapure water. RNaseA digestion was performed afterwards to remove any contaminant RNA and with achieving a final concentration of 20 µg.ml-1. Samples were incubated for 60 min at 65°C. To further concentrate the DNA and inactive

RNase, 10% volume of 3 M sodium acetate at pH 5.2 and 2.5 volumes of absolute ethanol were added, followed by a 1h incubation, and the tubes were centrifuged at 11 000 g during 30 min at room temperature. The supernatant was discarded, and the DNA pellet was rinsed with 500 µl of 70% ethanol. The DNA was then air-dried for 15 min before being resuspended in 40 µl of nuclease-free water. Large RNA was purified using the NucleoSpin RNA Blood kit from Macherey-Nagel according to the manufacturer's instructions, including proteinase K and on-column rDNAse digestions. RNA was eluted in 60 µl of nuclease-free water. 1 µl of obtained nucleic acids was quality-checked with an Agilent 2100 Bioanalyzer on a Small RNA chip, RNA 6000 Nano chip or DNA high sensitivity chip for small RNA, large RNA and DNA respectively, allowing quantification (Figure S1-2). The purity of the different fractions was analyzed using Nanodrop (ThermoFisher), and their concentrations were determined using Qubit (ThermoFisher), according to the user's manual (Figure S1A). Samples were frozen at -80°C until sequencing.

Extraction of extracellular proteins

For each individual, a 150 mg stool aliquot was gently resuspended in 900 µl of ice-cold 50 mM sodium-phosphate buffer, pH 7.4, supplemented with cOmplete Mini Protease Inhibitor Cocktail (Roche), and was processed the same way as the extracellular nucleic acid fractions, including centrifugation and filtration of the supernatant. Sodium deoxycholate (DOC) was added to a final concentration of 0.15% weight per volume to facilitate the precipitation of lowly abundant proteins. Following this, trichloroacetic acid (TCA) was added to a final concentration of 10% volume per volume and the sample was vortexed before a 30 min incubation on ice. A subsequent 15 min centrifugation at 10 000 g and 4°C allowed for proper protein precipitation. The pellet was then washed and centrifuged twice with 1.5 ml of ice-cold acetone before being dried under vacuum to remove any trace of any solvent. Finally, the protein pellet was resuspended in 100 µl of 50

mM sodium-phosphate buffer, pH 7.4, with the help of 2% w/v SDS and 15 sonication-bath cycles of 30s on / 30s off for complete solubilization. The protein concentration was determined by Qubit (according to the manufacturer's manual), as well as by a bicinchoninic acid assay (BCA) following the micro-plate protocol according to the manufacturer's instructions (ThermoFisher, averaging two technical duplicates for each sample (Figure S1B). Then, 150 µg of proteins were subjected to SDS-PAGE separation using a Criterion TM XT precast 12% Bis-Tris gel (Biorad). Electrophoresis was run on ice in MOPS buffer at 150 V for 1 hour 30 min. The gel was stained with Imperial Protein stain (Thermo Scientific) and each lane was cut into 11 pieces to isolate distinct protein sizes (Figure S3). The gel slices were frozen at -20°C and ready for tryptic digestion and mass spectrometry. A summary of the quantity of the extracellular macromolecules from the extractions is shown in Table S1-2.

Metabolite extractions – untargeted approach

To extract metabolites from stool samples, 500 µL of MilliQ water were added to 50 mg faecal matter. Then, samples were homogenized using a Precellys24 homogenizer (Bertin Technologies) using 5 ceramic beads (1.4 mm) and one 30 s cycle at 6000 rpm (0 to 5 °C). The homogenate was centrifuged at 21,000 *x* g for 5 min at 4 °C. Polar metabolites were extracted by transferring 50 µL of the supernatant into a new 0.5 mL Eppendorf tube and adding 20 µL of internal standard mix, consisting of U-13C5 ribitol (c = 150 µg/mL; Omicron Biochemicals), pentanedioic-d6 acid (c = 150 µg/mL; C/D/N Isotopes Inc.) and tridecanoic-d25 acid (c = 100 µg/mL; C/D/N Isotopes Inc.) in MilliQ water [1].This was followed by protein precipitation and a liquid-liquid extraction (LLE). First, 40 µL of the particulate-free homogenate was added to 160 µL Methanol. The mix was vortexed for 10 s, then incubated for 5 min at 2000 rpm and 15 °C (Eppendorf ThermoMixer Comfort), followed by a centrifugation at 21,000 *x* g for 5 min (15 °C). Then, 175 µL of the protein-free supernatant was added to 140 µL

Chloroform and 90 µL MilliQ water. The mixture was vortexed and incubated for 10 min at 2000 rpm and 15 °C. After centrifugation at 21,000 *x* g for 10 min (15 °C), 200 µL of upper phase containing polar metabolites were transferred in separate GC vials with micro insert and evaporated at -4°C for 4h, followed by 25 min at 25°C (Labconco CentriVap) [1]. The fractions were subsequently analyzed with gas chromatography-mass spectrometry (GC-MS). Metabolite derivatization was performed using a multi-purpose sampler (GERSTEL). Dried polar sample extracts were dissolved in 20 µL pyridine, containing 20 mg/mL of methoxyamine hydrochloride (Sigma-Aldrich), and incubated under shaking for 120 min at 45 °C. After adding 20 µL N-methyl-N-trimethylsilyl-trifluoroacetamide (MSTFA; Macherey-Nagel), samples were incubated for additional 30 min at 45 °C under continuous shaking.

GC-MS analysis was performed by using an Agilent 7890B GC – 5977A MS instrument (Agilent Technologies). A sample volume of 1 µL was injected into a Split/Splitless inlet, operating in split mode (10:1) at 270 °C. The gas chromatograph was equipped with a 5 m guard column + 30 m (I.D. 250 µm, film 0.25 µm) DB-35MS capillary column (Agilent J&W GC Column). Helium was used as carrier gas with a constant flow rate of 1.4 mL/min.

The GC oven temperature was held at 90 °C for 1 min and increased to 280 °C at 20 °C/min. Then, the temperature was increased to 325 °C and held for 4.5 min (post run time). The total run time was 15 min. The transfer line temperature was set constantly to 280 °C. The mass selective detector (MSD) was operating under electron ionization at 70 eV. The MS source was held at 230 °C and the quadrupole at 150 °C. Full scan mass spectra were acquired from *m/z* 70 to 600. All GC-MS chromatograms were processed using MetaboliteDetector, v3.220190704 [2]**.** Compounds were annotated by retention time and mass spectrum using an in-house mass spectral library. The following deconvolution settings were applied: Peak threshold: 5; Minimum peak height: 5; Bins per scan: 10; Deconvolution width: 5 scans; No baseline adjustment; Minimum 15 peaks per spectrum; No minimum required base peak intensity. The internal standards (U-13C5-ribitol and pentanedioic-d6 acid for polar fraction;

tridecanoic-d25 acid for nonpolar fraction) were added at the same concentration to every sample to correct for uncontrolled sample losses, analyte degradation during metabolite extraction and sensitivity drifts during measurements. The dataset was normalized by using the response ratio of the integrated peak area of the analyte and the integrated peak area of the internal standard. A heatmap on the measured intensities (Table S11) of the obtained metabolites is provided in Figure S4.

Metabolite extraction – short-chain fatty acids

To extract short-chain fatty acids from the stool samples, 500 µL of MilliQ water, containing 2-ethylbutyric acid (c = 2 mmol/L) as internal standard, were added to 50 mg of faecal matter. Then, samples were homogenized followed by centrifugation using the same parameters as described above. 200 µL of the supernatant were acidified with 10 µL of 37% hydrochloric acid, followed by an incubation step for 15 min at 2000 rpm and 15 °C (Eppendorf ThermoMixer Comfort). Then, 1 mL of diethyl ether were added. The samples were vortexed for 15 min at 2000 rpm and 15 °C. The upper organic phase was separated by centrifugation (5 min, 21,000 *x* g, 15 °C) and 900 µL were collected in a new reaction tube. A further 1 mL of diethyl ether was then added to the conditioned medium, and the tube was incubated, and its contents separated by centrifugation. Then, 900 µL of the organic phase were combined with the first extract, and 250 µL of this combined mixture were transferred into a GC glass vial with micro insert, in triplicate. For derivatization, 25 µL of N-tert-Butyldimethylsilyl-Nmethyltrifluoroacetamide (MTBSTFA) with 1% tert-Butyldimethylchlorosilane (TBDMSCl, Restek) was added, and the samples were incubated for a minimum of 2 h at room temperature. SCFAs were analyzed using GC-MS. Further measurement parameters have been previously described [3]. For precise quantification, measurements were performed in selected ion monitoring mode. GC-MS chromatograms were processed using Agilent MassHunter Quantitative Analysis (ver. B.08.00, Build 8.0.598.0). Target

compounds were identified by retention time and ion ratios using an in-house mass spectral library. The data set was normalized by using the response ratio of the integrated peak area of the target compound and the integrated peak area of the internal standard. Absolute concentrations were determined using calibration curves made of authentic standards. The obtained measurements are shown in Table S10 and summarized in Figure 2C.

Metabolite extractions – bile acids

To extract metabolites from stool samples, 500 µl of MilliQ water were added to 50 mg faecal matter. Then, samples were homogenized followed by centrifugation using the same parameters as described above. To extract bile acids, a total volume of 100 µl of the supernatant was added to 100 µl MilliQ water, incl. internal standards (cholic-d4 Acid, deoxycholic-d4 Acid, lithocholic-d4 Acid, glycocholic-d4 Acid; c = 1 µg/ml each; C/D/N Isotopes) and vortexed. Following this, 100 µl of the mixture was introduced into 500 µl of ice-cold acetonitrile and incubated for 5 min at 4°C under shaking followed by centrifugation at 21,000 *x* g for 5 min. The supernatant was collected and filtered using a Phree phospholipid removal plate (Phenomenex). 500 µl were concentrated under vacuum (SpeedVac, Labconco) at -4°C overnight. The resultant dry residues were reconstituted in 100 µl of ACN/MeOH/H2O mixture (1:1:2) and transferred to LC vials for LC-HRMS analysis. The analyses were performed using a Dionex Ultimate3000 LC coupled to a Thermo Q Exactive MS instrument. Chromatography was carried out with a Waters ACQUITY UPLC CSH C18 1.7 µm (2.1 mm x 100 mm) column and VanGuard pre-column (2.1mm x 5 mm) maintained at 60 °C. The autosampler was kept at 4 °C. The flow rate was set to 0.3 ml/min and the mobile phases consisted of 5 mM ammonium acetate in water (Eluent A) and 50:50 Methanol/Acetonitrile (Eluent B). The run consisted of linear gradient form 40% B to 65% B over 6 min, followed by a linear gradient to 95% B over 3 min, isocratic delivery of 95% B for 9 min,

and a re-equilibration phase on starting conditions with 40%B for 7 min. The injection volume was 3 µl. All the MS experiments were performed using electrospray ionization in negative mode (-ESI). The source and ion transfer parameters applied were as follows: spray voltage 3.5 kV. The sheath gas, aux gas, the capillary temperature and the heater temperature were maintained at 45, 10 (arbitrary units), 300°C and 300 °C, respectively. The S-Lens RF level was set at 50. The Orbitrap mass analyser was operated at a resolving power of 35 000 in full-scan mode (scan range: m/z 100…620; automatic gain control (AGC) target: 3e6; injection time: 100 ms). Mass spectrometric data were acquired with Thermo Xcalibur software (Version 4.1.31.9) and analysed with TraceFinder (Version 4.1). For unambiguous annotation of the target compounds, an in-house library with retention times and MS/MS spectra were used. The identity of all bile acids was confirmed by MS/MS and standard addition experiments. Target peak area was normalized by corresponding internal standard peak area. Absolute concentrations were determined using calibration curves made of authentic standards. The obtained measurements are shown in Table S10 and summarized in Figure 2C.

Metagenomics

200-300 ng of intracellular DNA was sheared using Bioruptor NGS (Diagenode, UCD300) with 30s ON and 30s OFF for 20 cycles. The sequencing libraries were prepared using TruSeq Nano DNA library preparation kit (Illumina, FC-121-4002) using the protocol provided with the kit. The libraries were prepared considering a 350 bp average insert size. Prepared libraries were quantified using Qubit (Invitrogen) and the quality was checked on a Bioanalyzer (Agilient). Sequencing was performed on a NextSeq500 (Illumina) instrument using 2x150 bp read length at the LCSB Sequencing Platform.

For the extracellular DNA fraction, 150 ng of DNA per sample was used for library preparation using the TrueSeq Nano DNA Kit (Illumina). The libraries were prepared considering 350 bp average insert size and sequenced similarly to the intracellular DNA.

Metatranscriptomics

2 µg of large RNA per sample was subjected to rRNA removal (Ribo-Zero rRNA Removal Kit, Illumina) before library preparation (True stranded mRNA LT Kit, Illumina). The average insert size was 400 bp. 500 ng of small RNA per sample was used for library preparation using NEBNext Multiplex Small RNA Library Prep Kit for Illumina (NEB), including AMPure XP magnetic beads-based size-selection of small RNA. The average size was 190 bp.

Sequencing

The insert size and the quality of libraries were checked with an Agilent 2100 Bioanalyzer and quantified using the Qubit dsDNA HS assay kit. Libraries were diluted to 4 nM each, pooled, denatured and sequenced for 2 × 150 cycles on a NextSeq500 (Illumina) instrument according to the manufacturer’s instructions, except for the sRNA libraries that was sequenced at 1x75 bp read length. The raw sequence libraries are deposited in the European Nucleotide Archive (ENA) at EMBL-EBI under accession number PRJEB44766.

Metaproteomics

Extraction of proteins from the gel bands and subsequent digestion was performed using an in-gel digestion protocol described previously [4]. Briefly, the excised gel pieces were rinsed with two successive additions of 1 ml HPLC-grade water. Gel pieces were de-stained using 50 mM acetonitrile/50 mM ammonium bicarbonate (1:1, vol/vol) and the gel pieces were pulverized with a pestle before incubation for 30 min. 100% acetonitrile was added to dehydrate and shrink the de-stained gel fragments, and then as much liquid as possible was removed without disturbing any of the gel fragments to prepare fragments for digestion. Proteomics-grade trypsin was dissolved to 13 ng/l in a 10 mM ammonium bicarbonate buffer containing 10% (vol/vol) acetonitrile. 100 l of the trypsin solution was added to the cover the gel fragments and incubated at 4°C for 2 hours. After rehydration, the gel fragments were incubated and shaken overnight at 37°C and 600 rpm. Following digestion, peptides were extracted from the gel matrix using 5% formic acid/acetonitrile solution in a ratio of 1:2 sample volume: extraction buffer and incubated for 15 min at 37°C. The supernatant containing the extracted peptides was transferred to a clean tube and dried down using a SpeedVac centrifuge. The digested peptides were resuspended in solvent A (95% water/5% acetonitrile/0.1% formic acid) and peptide concentration was measured by a NanoDrop OneC (ThermoScientific) using absorbance at 205 nm. The obtained proteolytic peptides were analyzed via an automated nanospray Vanquish LC-QExactive-Plus Orbitrap mass spectrometer system outfitted with a 100 μm ID trapping column coupled to an in-house pulled 75 μm ID analytical column. Both the trapping column and analytical columns were packed with 5μm Kinetex C-18 RP resin (Phenomenex) to 6 cm and 25 cm, respectively. For each gel segment, 10μL of digested peptides was loaded, desalted, separated and analyzed with the following parameters: loading and desalting in 100% solvent A for 30 min, separation with a 65 min linear gradient up to 25% solvent B (30% water/70% acetonitrile/0.1% formic acid), another increase to 50% solvent B for 5 min, re-equilibration back to 100% solvent A for 5 min, and a wash in 100% solvent A for 15 min. All eluting peptides were measured and sequenced under data-dependent acquisition on the Q Exactive mass spectrometer. An overview of the quantities

of extracted proteins from each sample is presented in Table S3. The raw MS files are deposited in the MassIVE, ProteomeXchange and PRIDE databases under the experiment accession numbers MSV000086973 and PXD024472, respectively.

Bioinformatic analyses

Metagenomics and metatranscriptomics

Integrated analysis of the metagenomic and metatranscriptomic data was performed using IMP (v1, default settings) [5]. First, adapters from all sequenced samples (int-DNA, int-RNA, ex-DNA, ex-lRNA and ex-sRNA) were removed with Cutadapt (v.2.4) [6] and rRNA sequences were filtered from all RNA fractions (int-RNA, ex-lRNA and ex-sRNA) by SortMeRNA (v2.0, default settings) [7] to obtain putative mRNA sequences. To obtain a meta-genome and meta-transcriptome (MG-MT) reference for each individual, int-DNA and int-RNA reads were co-assembled into contigs by iterative alignment using the BWA-MEM aligner (v0.7.17) [8]. Co-assembled contigs were binned using two different algorithms MetaBAT 2 (v.2.12.1) [9] and MaxBin 2.0 (v2.2.6) [10] and their results were refined with DAS Tool (v0.9.24, default settings) [11] to further optimize the binning. Taxonomical annotation of those bins was done with GTDB-Tk (v0.3.2, default settings) [12]. Open Reading Frames (ORFs) as a proxy for genes were inferred from these MG-MT references using Prokka (v1.13.7, default settings) [13] and functional annotation of those predicted genes was done with eggNOG-mapper (v1.0.3) [14] (Table S8). In addition to the discussed IMP pipeline, gene counts for all fractions (int-DNA, int-RNA, ex-DNA, ex-lRNA and ex-sRNA) were calculated using featureCounts (v1.6.4, default settings) [15], after aligning the reads to the references with BWA-MEM [8]. Taxonomic classification for all fractions was performed on the co-assembled contigs using Kraken2 (v2.0.8) [16] against its standard default database (Figure 2A, Table S6). Functional annotation was obtained according to Clusters of Orthologous Groups

(COG) (Figure 2B, Table S7). Furthermore, Shannon diversities (Figure S4, Table S4) and concordance correlation coefficients (Table S5) [17] were calculated on the obtained taxonomic and functional annotation for all fractions. Subsequently to integrate the findings, all data from the individual fractions were grouped as represented in Fig. 2, by linking the taxonomic and functional affiliations across fractions from each individual. For the comparison of the various biomolecular fractions at the nucleotide level, Sourmash was used to created *signatures* using the default K-mer value of 31 [18]. The figures for Sourmash were generated by the tool using its inbuilt functions. All other plots aside from the Upset plots were generated using the R package ggplot2 [19]. The Upset plots were generated using the ComplexUpset package in R [20].

Metaproteomics

A concatenated database was created for each sample using the co-assembles contigs obtained from the previously described intracellular omics analysis of each individual, the human proteome, common contaminants, and reversed sequences to assess false discovery rates (FDR). Experimental MS/MS spectra were searched using the MyriMatch [21] search algorithm (v2.1.138) with the following settings: peptide-spectrum matches (PSM) were required to be fully tryptic with up to two missed cleavages, static cysteine modification of 57.0214 Da and dynamic oxidation modifications of 15.9949 Da on methionine residues were included in the searches. Resulting peptide-spectrum matches were filtered at a final peptide-level FDR <1% using IDPicker [22] (v3.1.642.0). The proteome database was clustered at 100% amino acid sequence identity, and peptides were reassigned to protein groups based on the clustering. The peptide chromatographic AUC (area under the curve) intensities were used to assemble proteins by summing only peptide intensities that uniquely mapped to one protein group. For each protein group, the abundances of each gel band fraction were summed to obtain sample-level

abundances. All four sample-specific databases used for searching were clustered together at 100% amino acid sequence identity. Sample-specific protein groups were assigned to experiment-wide protein groups based on the clustering to compare protein abundances across all four individuals more easily. The resulting protein group-level intensities were log-transformed, normalized at the sample level by LOESS, and standardized across the entire dataset by median absolute deviation (MAD) and median centering using InfernoRDN [23] (Table S9).

Metabolomics

The functional annotations of the predicted genes obtained through eggNOG-mapper were manually compared to the metabolites identified in the metabolite fraction.

Supplementary References

1. Glaab E, Trezzi J-P, Greuel A, Jäger C, Hodak Z, Drzezga A, et al. Integrative analysis of blood metabolomics and PET brain neuroimaging data for Parkinson's disease. *Neurobiol Dis*. 2019 Apr 1; **124**: 555–62.

2. Hiller K, Hangebrauk J, Jäger, C, Spura J, Schreiber K, Schomburg D. MetaboliteDetector: Comprehensive Analysis Tool for Targeted and Nontargeted GC/MS Based Metabolome *Analysis. Anal Chem*. 2009; **81**, 9, 3429–3439

3. Greenhalgh K, Ramiro-Garcia J, Heinken A, Ullmann P, Bintener T, Pacheco MP, et al. Integrated In Vitro and In Silico Modeling Delineates the Molecular Effects of a Synbiotic Regimen on Colorectal-Cancer-Derived Cells. *Cell Rep.* 2019 Apr 30; **27(5)**: 1621-1632.e9.

4. Shevchenko A, Tomas H, Havli J, Olsen J, Mann M. In-gel digestion for mass spectrometric characterization of proteins and proteomes. *Nat Protoc.* 2006; **1(6)**: 2856–60.

5. Narayanasamy S, Jarosz Y, Muller EEL, Heintz-Buschart A, Herold M, Kaysen A, et al. IMP: a pipeline for reproducible reference-independent integrated metagenomic and metatranscriptomic analyses. *Genome Biol.* 2016 Dec; **17(1)**: 260.

6. Martin M. Cutadapt removes adapter sequences from high-throughput sequencing reads. *EMBnet.journal*. 2011 May 2; **17(1)**: 10–2.

7. Kopylova E, Noé L, Touzet H. SortMeRNA: fast and accurate filtering of ribosomal RNAs in metatranscriptomic data. *Bioinformatics*. 2012; **28(24)**: 3211–7.

8. Li H, Durbin R. Fast and accurate short read alignment with Burrows–Wheeler transform. *Bioinformatics*. 2009 Jul 15; **25(14)**: 1754–60.

9. Kang DD, Li F, Kirton E, Thomas A, Egan R, An H, et al. MetaBAT 2: an adaptive binning algorithm for robust and efficient genome reconstruction from metagenome assemblies. *PeerJ*. 2019 Jul 26; **7**: e7359.

10. Wu Y-W, Simmons BA, Singer SW. MaxBin 2.0: an automated binning algorithm to recover genomes from multiple metagenomic datasets. *Bioinformatics*. 2016 Feb 15; **32(4)**: 605–7.

11. Sieber CMK, Probst AJ, Sharrar A, Thomas BC, Hess M, Tringe SG, et al. Recovery of genomes from metagenomes via a dereplication, aggregation and scoring strategy. *Nat Microbiol*. 2018 Jul; **3(7)**: 836–43.

12. Chaumeil P-A, Mussig AJ, Hugenholtz P, Parks DH. GTDB-Tk: a toolkit to classify genomes with the Genome Taxonomy Database. *Bioinformatics*. 2020 Mar 1; **36(6)**: 1925–7.

13. Seemann T. Prokka: rapid prokaryotic genome annotation. *Bioinformatics*. 2014 Jul 15; **30(14)**: 2068–9.

14. Huerta-Cepas J, Forslund K, Coelho LP, Szklarczyk D, Jensen LJ, von Mering C, et al. Fast Genome-Wide Functional Annotation through Orthology Assignment by eggNOG-Mapper. *Mol Biol Evol.* 2017 Aug 1; **34(8)**: 2115–22.

15. Liao Y, Smyth GK, Shi W. featureCounts: an efficient general purpose program for assigning sequence reads to genomic features. *Bioinforma Oxf Engl*. 2014 Apr 1; **30(7)**: 923–30.

16. Wood D, Lu J, Langmead B. Improved metagenomic analysis with Kraken 2. *Genome Biol*. 2019; **20**: 257.

17. Lin LI-K. A Concordance Correlation Coefficient to Evaluate Reproducibility. *Biometrics*. 1989; **45(1)**: 255–68.

18. Pierce NT, Irber L, Reiter T *et al.* Large-scale sequence comparisons with *sourmash*. *F1000Research* 2019, **8**: 1006.

19. Wickham H. ggplot2: Elegant Graphics for Data Analysis. Springer; 2016. 266 p.

20. Michał Krassowski. (2020). krassowski/complex-upset. Zenodo. http://doi.org/10.5281/zenodo.3700590

21. Tabb D, Fernando C, Chambers M. MyriMatch: highly accurate tandem mass spectral peptide identification by multivariate hypergeometric analysis. *J Proteome Res.* 2007 Jan 18; **6**: 654–61.

22. Ma Z-Q, Dasari S, Chambers MC, Litton MD, Sobecki SM, Zimmerman LJ, et al. IDPicker 2.0: Improved Protein Assembly with High Discrimination Peptide Identification Filtering. *J Proteome Res*. 2009 Aug 7; **8(8)**: 3872–81.

23. Polpitiya AD, Qian WJ, Jaitly N, Petyuk VA, Adkins JN, Camp DG 2nd, et al. DAnTE: a statistical tool for quantitative analysis of -omics data. *Bioinformatics*. 2008; **24(13)**: 1556–8.

Supplementary Figures

Provided as separate files:

Figure S1: Illustration depicting the integrated workflow, whereby the iterative metagenomic and metatranscriptomic co-assembly is used to search the metaproteomic spectra. The metabolomic data is subsequently used for downstream integration and visualisation.

Figure S2: An example overview for the integrated multi-omics analyses depicting the presence and relative abundance of the individual omic data for the L-threonine → Propionic acid pathway. The values represent the mean of the relative abundances across Individuals 1 to 4, whereby the check marks indicate the detection within the respective dataset.

Figure S3: Concentrations (ng per mg stool) of the different extracellular nucleic acid (A) and protein (B) fractions (logarithmic scale), obtained by Bioanalyzer, Qubit, Nanodrop and/or BCA analysis. Error bars represent the standard deviation on four independent samples. ex-sRNA: extracellular small RNA; ex-lRNA: extracellular large RNA; ex-DNA: extracellular DNA; ex-Prot: extracellular proteins.

Figure S4: Electropherograms from the of extracted extracellular nucleic acids. A. Extracellular DNA (DNA high sensitivity chip). B. Extracellular large RNA (RNA 6000 Nano chip). C.

Extracellular small RNA (Small RNA chip). Each extraction was performed for four individuals with a buffer-only control and 1 µl of each sample was run on the Agilent 2100 Bioanalyzer.

Figure S5: SDS-PAGE gel image of extracted extracellular protein fractions. A. Extracellular proteins profiles from Individual 1 to 4 (150 µg/lane) and an extraction blank are presented from left to right. The figure is a montage of two gels, run under the same conditions, separated by a solid line. One lane was cut out from the gel, symbolized by a dashed line. B. Full length uncropped gel for Individual 1.

Figure S6: Comparison of the average relative abundances of DNA/RNA/Prot in our extracellular extractions (A) and the repartition expected inside a bacterial cell [20] (B). A. The obtained composition of the extracted extracellular mass (DNA, RNA and Prot) in %, before experimental rRNA depletion. Percentages are relative to the original stool mass used for extraction. B. Average intracellular composition (DNA, RNA and Prot) of a bacterial cell in % dry weight. Figure is adapted from estimations based on experimental data obtained in *E. coli* [20] where only the DNA/RNA/Prot relative abundances were retained. ex-sRNA: extracellular small RNA; ex-lRNA: extracellular large RNA.

Figure S7: Relative abundance (%) of Roseburia spp. in each extracted fraction for each individual separately.

Figure S8: Shannon diversities of the taxa (A) and functions (B) for each ome and individual. More variation can be observed between the individual diversities of the taxa within and between the different omes. Individual diversities of the functions are more similar within and between the different omes, with ex-lRNA and ex-sRNA being divergent. int-DNA: intracellular DNA; ex-DNA: extracellular DNA; int-RNA: intracellular RNA; ex-sRNA: extracellular small RNA; ex-lRNA: extracellular large RNA; ex-Prot: extracellular proteins.

Figure S9: Sourmash plots assessing the overlap between the intra- and extracellular fractions of the different biomolecules for each individual separately. Heatmap of the nucleic acid signatures for the intra- and extracellular fractions demonstrating the hierarchical clustering based on Ward's algorithm and average linkage, between fractions for each individual.

Figure S10: Upset plots indicating the overlap between the taxonomic affiliations for each fraction within each individual. The bar plots represent the relative abundance of the taxa overlapping across the fractions, while the intersect sizes indicate the number of common taxa.

Figure S11: Functional overlap. The functional overlap for each fraction for each individual are shown in the Upset plots. The bar plots represent the relative abundance of the functions overlapping across the fractions, while the intersect sizes indicate the number of common functions. Abbreviations of the functional categories: A: RNA processing and modification; B: Chromatin structure and dynamics; C: Energy production and conversion; D: Cell cycle control: cell division: chromosome partitioning; E: Amino acid transport and metabolism; F: Nucleotide transport and metabolism; G: Carbohydrate transport and metabolism; H: Coenzyme transport and metabolism; I: Lipid transport and metabolism; J: Translation: ribosomal structure and biogenesis; K: Transcription; L: Replication: recombination and repair; M: Cell wall/membrane/envelope biogenesis; N: Cell motility; O: Post-translational modification: protein turnover and chaperones; P: Inorganic ion transport and metabolism; Q: Secondary metabolites biosynthesis: transport and catabolism; S: Function unknown; T: Signal transduction mechanisms; U: Intracellular trafficking: secretion and vesicular transport; V: Defense mechanisms; Z: Cytoskeleton.

Figure S12: Relative abundance (%) of *Methanobrevibacter smithii* in each extracted fraction for each individual separately.

Figure S13: Heatmap of the relative abundances of metabolites for each individual measured in an untargeted GC-MS. Low abundances are indicated in light blue; higher abundances are shown in dark blue and red.

Supplementary Tables

Provided as separate files:

Table S1: Quantities of the extracted extracellular macromolecules relative to the stool mass from each individual.

Table S2: Quantities of the proteins extracted from each sample.

Table S3: Measurements on the purified fractions of the blank samples. An x indicates that the analysis was not performed on that particular sample.

Table S4: PERMANOVA-based estimations of significantly different taxa between fractions, within individuals. The adjusted *p*-value is considered to be significant when below 0.05.

Table S5: Mean (±SD) Shannon diversities on the taxonomic and functional composition for each ome across the four individuals.

Table S6: Mean (±SD) concordance correlation coefficients on the taxonomic and functional composition for each ome across the four individuals.

Table S7: PERMANOVA-based estimations of significantly different functions when comparing fractions, within individuals. A *p*-value less than 0.05 is considered to be significant.

Table S8: Taxonomic annotation up to species level and corresponding number of reads for each sample. Undetermined annotations are represented by the first letter of the corresponding level. Figure 2A is based on this information. (Available online via Figshare; DOI: 10.6084/m9.figshare.c.5694595.v1).

Table S9: Functional annotation (COG) and corresponding number of reads for each sample. Figure 2B is based on this information.

Table S10: Functional annotation, corresponding counts, genes and taxonomy of the obtained contigs for every fraction from each individual. (Available online via Figshare; DOI: 10.6084/m9.figshare.c.5694595.v1).

Table S11: Raw and normalized protein intensities for each individual obtained after LC-MS/MS based metaproteomics analysis. (Available online via Figshare; DOI: 10.6084/m9.figshare.c.5694595.v1).

Table S12: Concentrations of the SCFAs (in µmol/L) and BAs (in µg/L) measured during targeted GC-MS and LC-HRMS, respectively. Dynamic range for the SCFA and BA measurements ranges from 10 to 4 000 µmol/L and from 50 to 4 000 µg/L, respectively.

Table S13: Intensities measured during an untargeted metabolomics analysis using GC-MS.

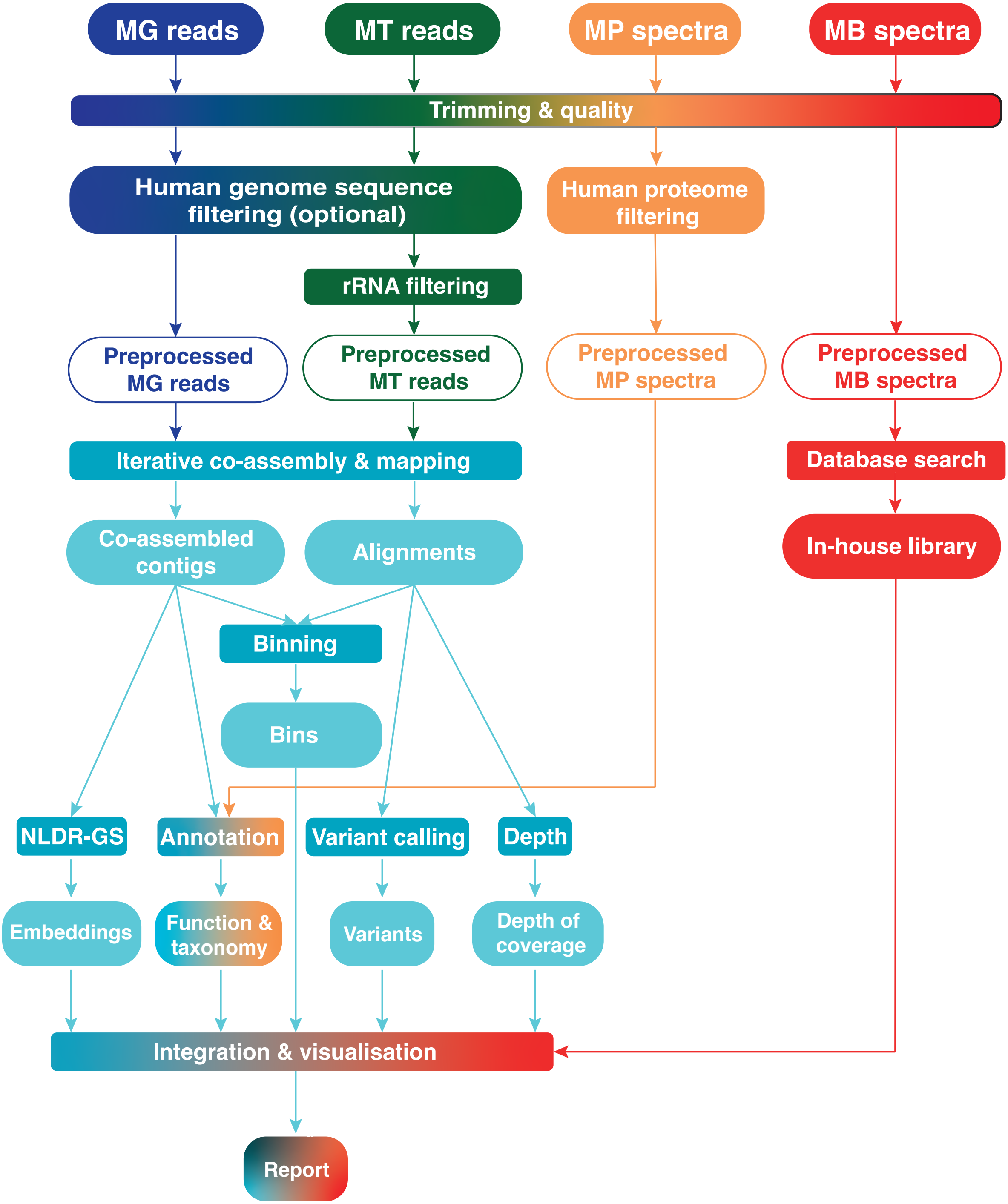
MG reads
MT reads
MP spectra
MB spectra
Trimming & quality
Human genome sequence filtering (optional)
Human proteome filtering
rRNA filtering
Preprocessed MG reads
Preprocessed MT reads
Preprocessed MP spectra
Preprocessed MB spectra
Iterative co-assembly & mapping
Database search
Co-assembled contigs
Alignments
In-house library
Binning
Bins
NLDR-GS
Annotation
Variant calling
Depth
Embeddings
Function & taxonomy
Variants
Depth of coverage
Integration & visualisation
Report

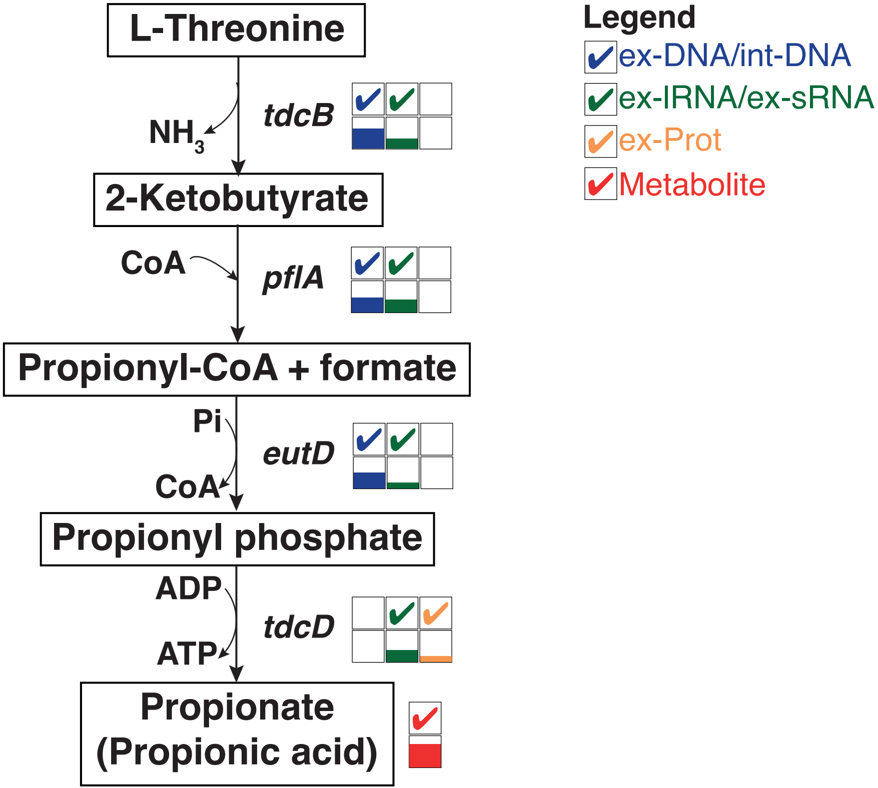
L-Threonine
NH$_3$
tdcB
2-Ketobutyrate
CoA
pflA
Propionyl-CoA + formate
Pi
CoA
eutD
Propionyl phosphate
ADP
ATP
tdcD
Propionate
(Propionic acid)
Legend
ex-DNA/int-DNA
ex-lRNA/ex-sRNA
ex-Prot
Metabolite

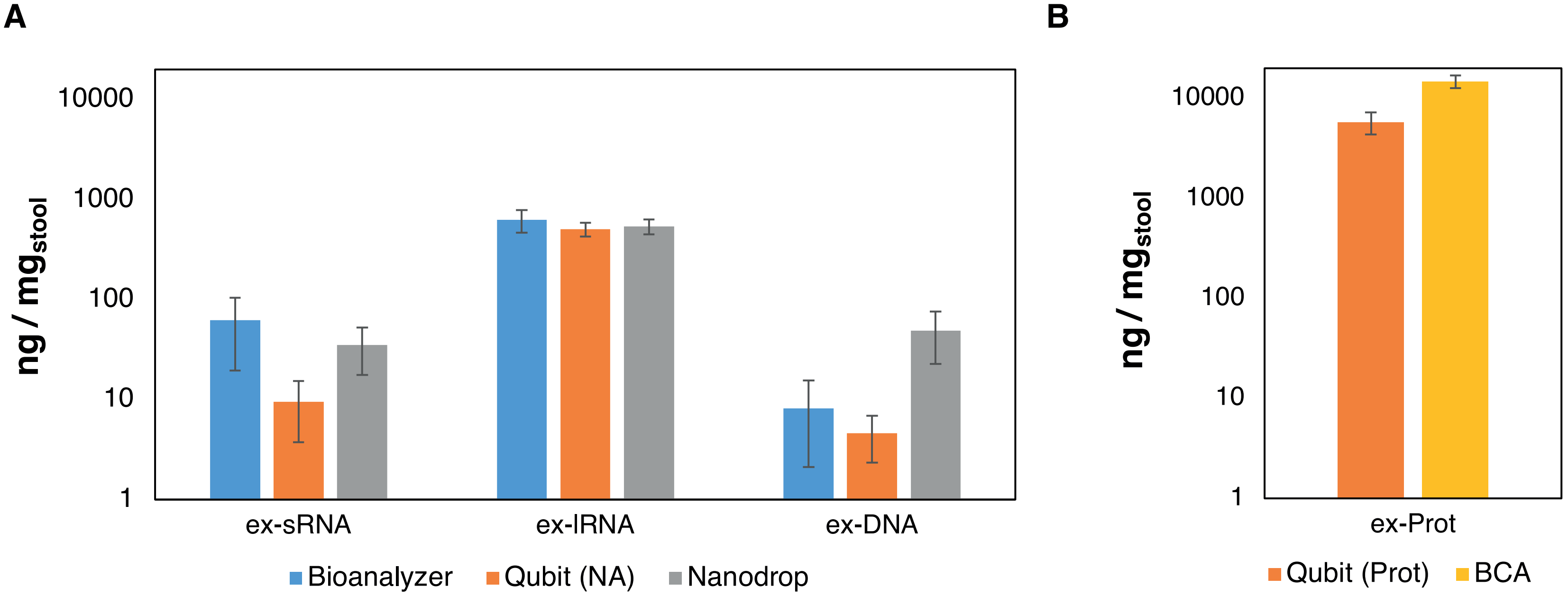
A
B
ng / mg_{stool}
10000
1000
100
10
1
ex-sRNA
ex-lRNA
ex-DNA
Bioanalyzer
Qubit (NA)
Nanodrop
ng / mg_{stool}
10000
1000
100
10
1
ex-Prot
Qubit (Prot)
BCA

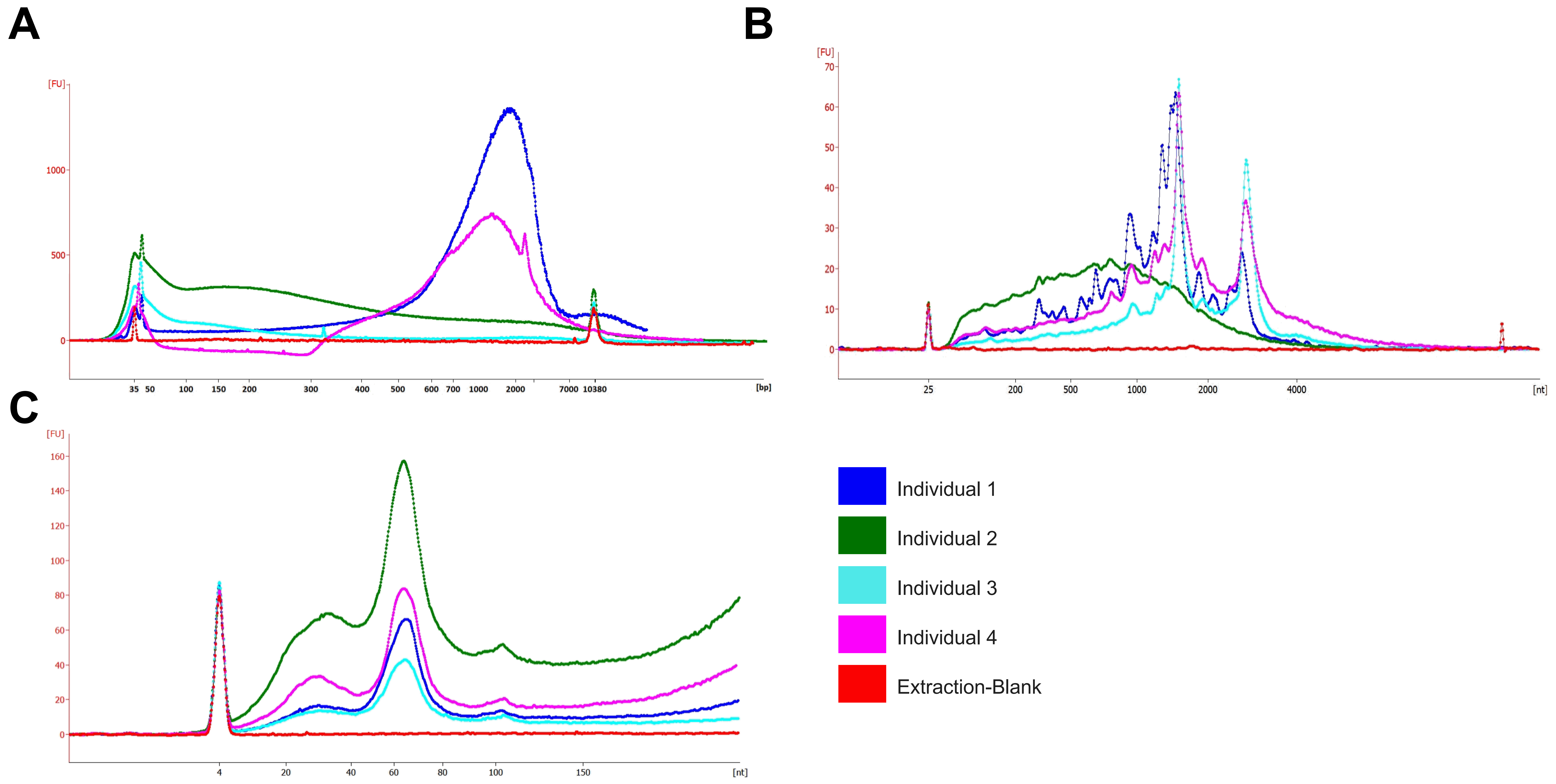

A
B
C
[FU]
[bp]
[nt]
35 50 100 150 200 300 400 500 600 700 1000 2000 7000 10380
25 200 500 1000 2000 4000
4 20 40 60 80 100 150
Individual 1
Individual 2
Individual 3
Individual 4
Extraction-Blank

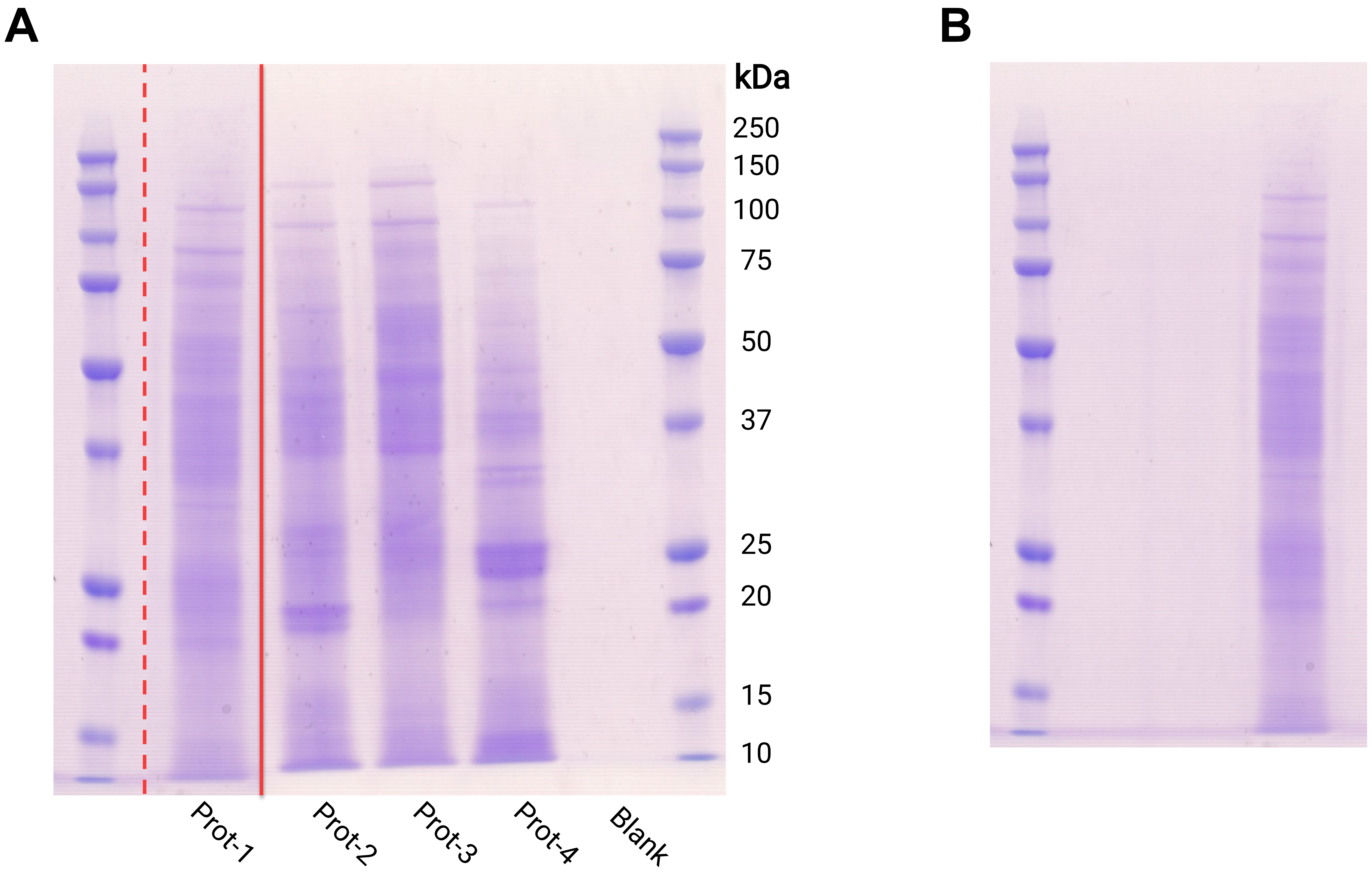
A
B
kDa
250
150
100
75
50
37
25
20
15
10
Prot-1
Prot-2
Prot-3
Prot-4
Blank

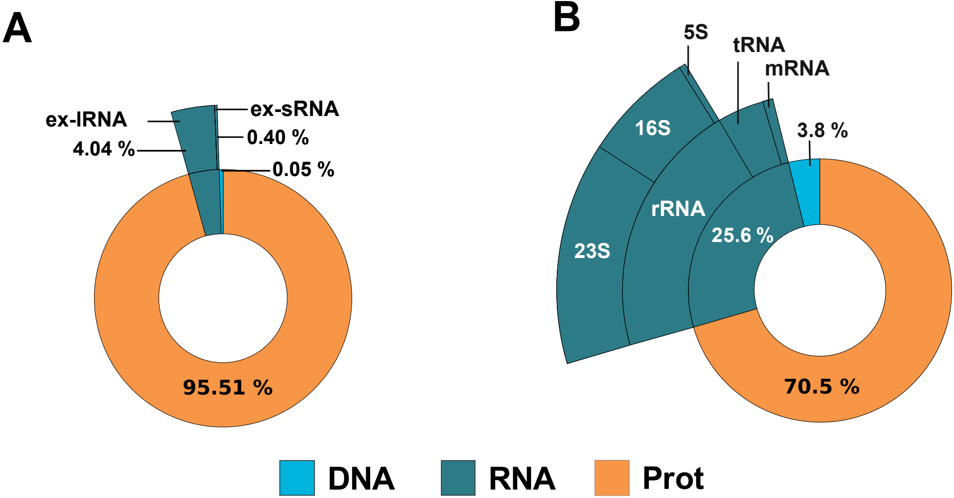
A
ex-lRNA
4.04 %
ex-sRNA
0.40 %
0.05 %
95.51 %
B
5S
tRNA
mRNA
16S
3.8 %
rRNA
25.6 %
23S
70.5 %
DNA
RNA
Prot

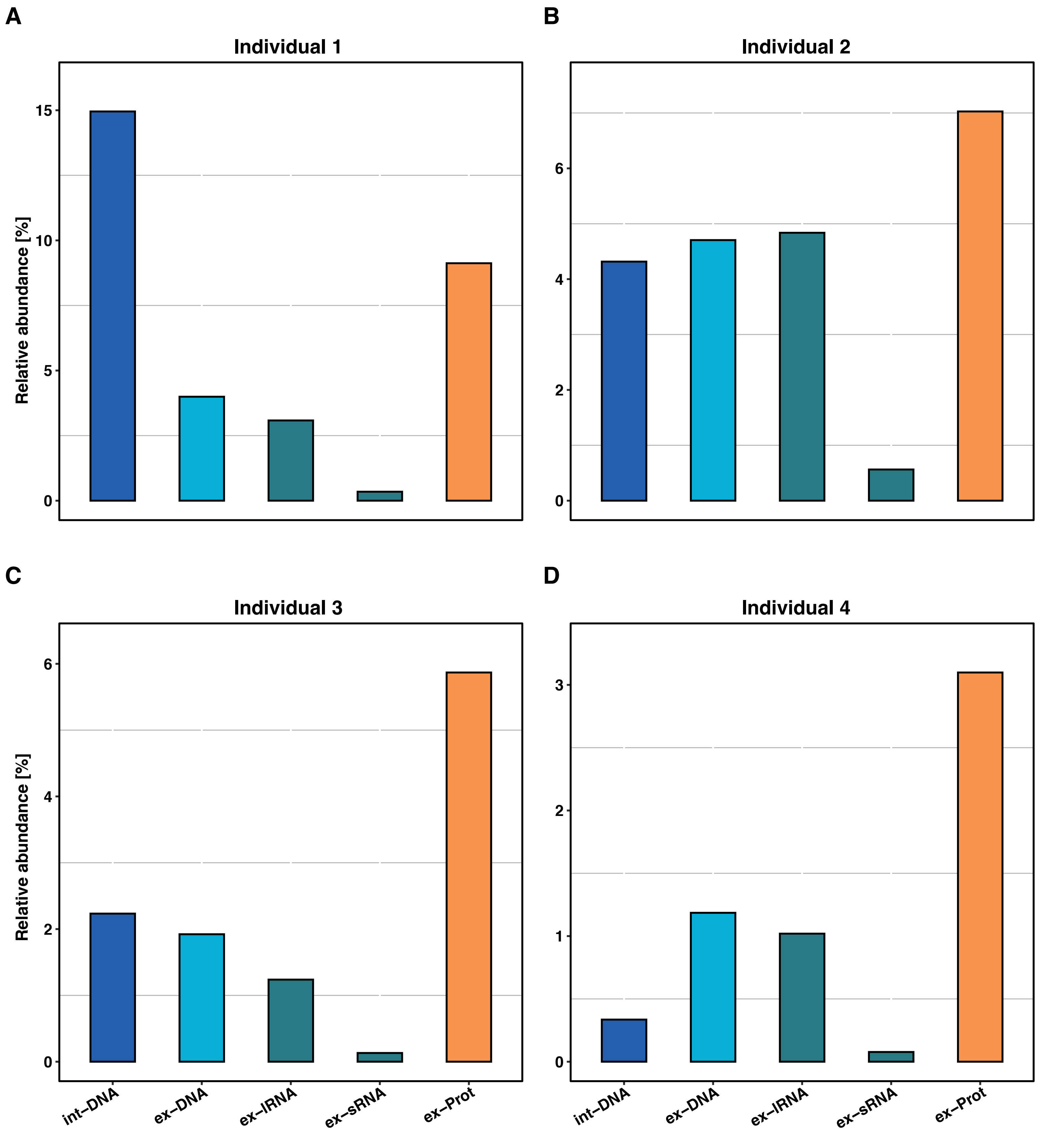
A
Individual 1
Relative abundance [%]
0
5
10
15
B
Individual 2
0
2
4
6
C
Individual 3
Relative abundance [%]
0
2
4
6
D
Individual 4
0
1
2
3
int–DNA
ex–DNA
ex–lRNA
ex–sRNA
ex–Prot

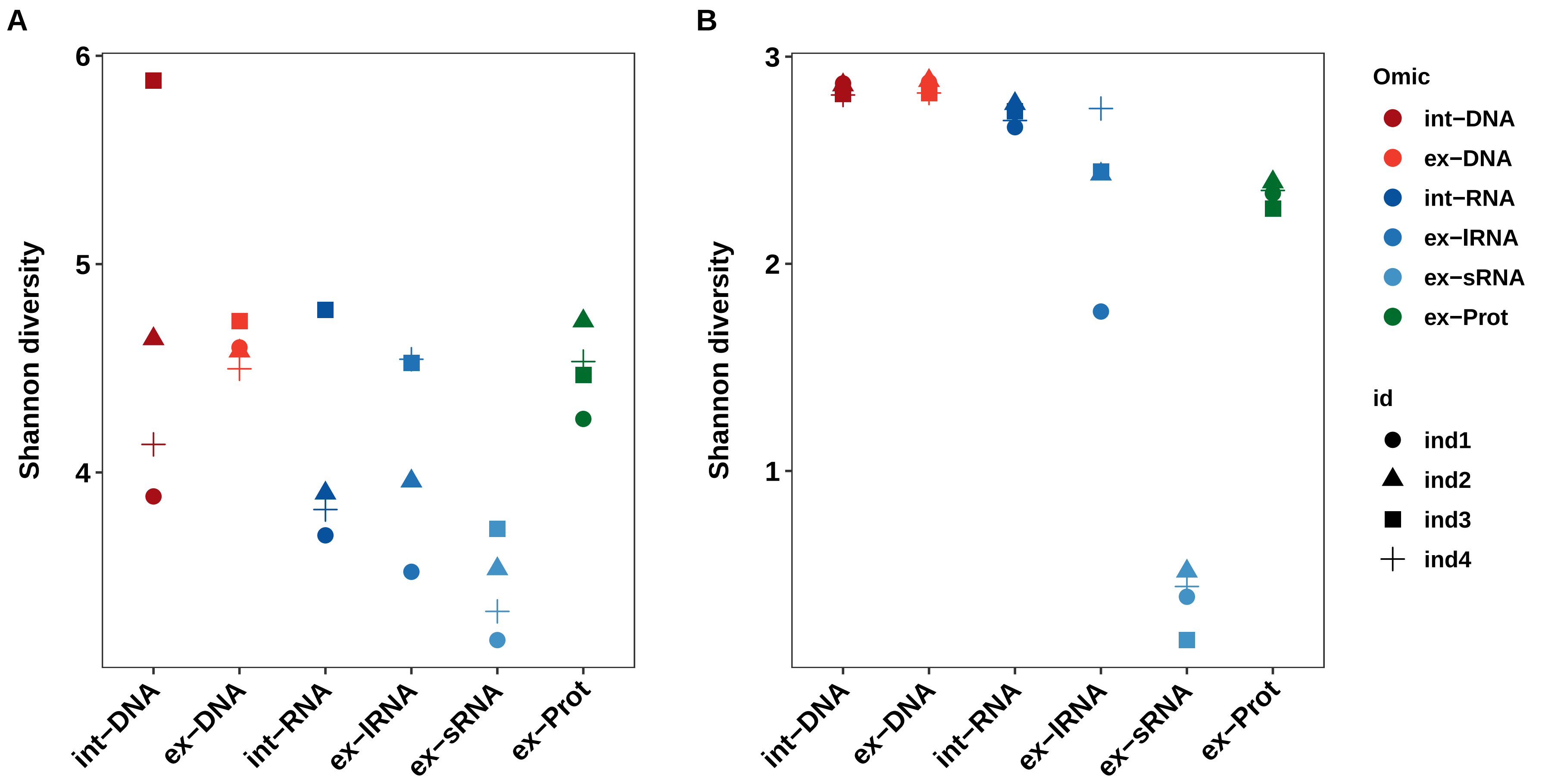
A
B
Shannon diversity
6
5
4
3
2
1
int−DNA
ex−DNA
int−RNA
ex−lRNA
ex−sRNA
ex−Prot
Omic
id
ind1
ind2
ind3
ind4

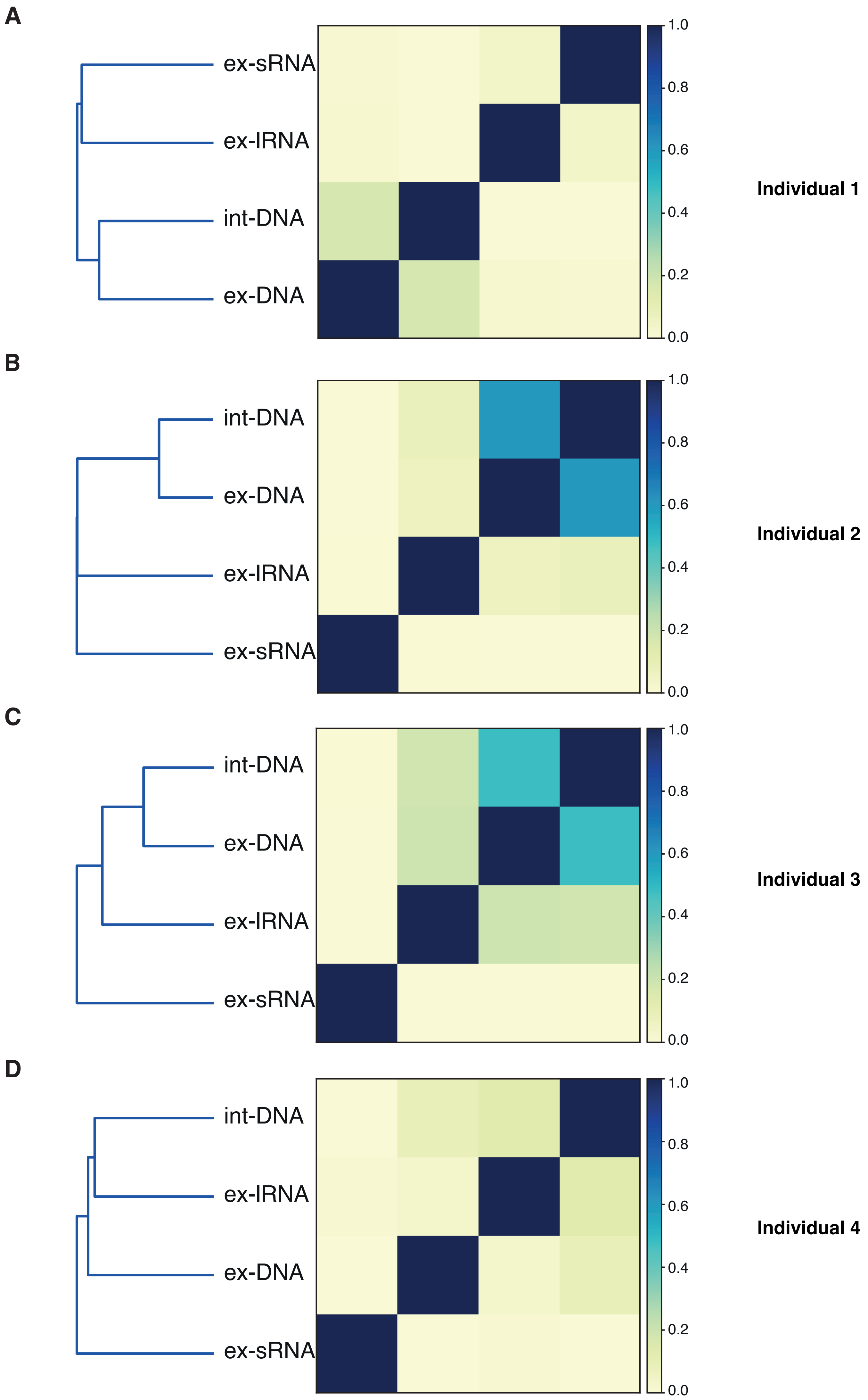
A
ex-sRNA
ex-lRNA
int-DNA
ex-DNA
Individual 1
B
int-DNA
ex-DNA
ex-lRNA
ex-sRNA
Individual 2
C
int-DNA
ex-DNA
ex-lRNA
ex-sRNA
Individual 3
D
int-DNA
ex-lRNA
ex-DNA
ex-sRNA
Individual 4
1.0
0.8
0.6
0.4
0.2
0.0

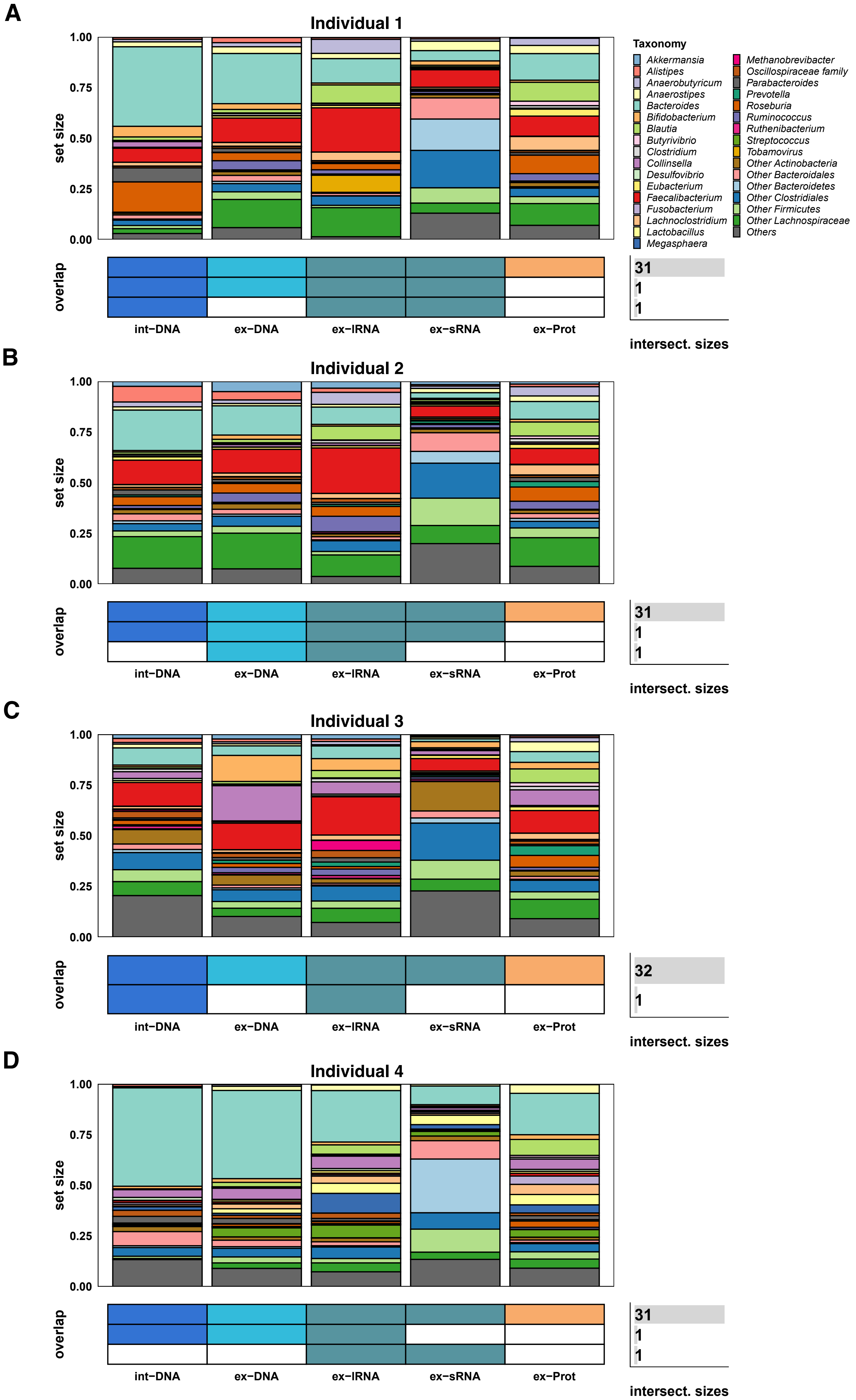
A
Individual 1
B
Individual 2
C
Individual 3
D
Individual 4
1.00
0.75
0.50
0.25
0.00
set size
overlap
int−DNA
ex−DNA
ex−lRNA
ex−sRNA
ex−Prot
intersect. sizes
31
1
32
Taxonomy
Akkermansia
Alistipes
Anaerobutyricum
Anaerostipes
Bacteroides
Bifidobacterium
Blautia
Butyrivibrio
Clostridium
Collinsella
Desulfovibrio
Eubacterium
Faecalibacterium
Fusobacterium
Lachnoclostridium
Lactobacillus
Megasphaera
Methanobrevibacter
Oscillospiraceae family
Parabacteroides
Prevotella
Roseburia
Ruminococcus
Ruthenibacterium
Streptococcus
Tobamovirus
Other Actinobacteria
Other Bacteroidales
Other Bacteroidetes
Other Clostridiales
Other Firmicutes
Other Lachnospiraceae
Others

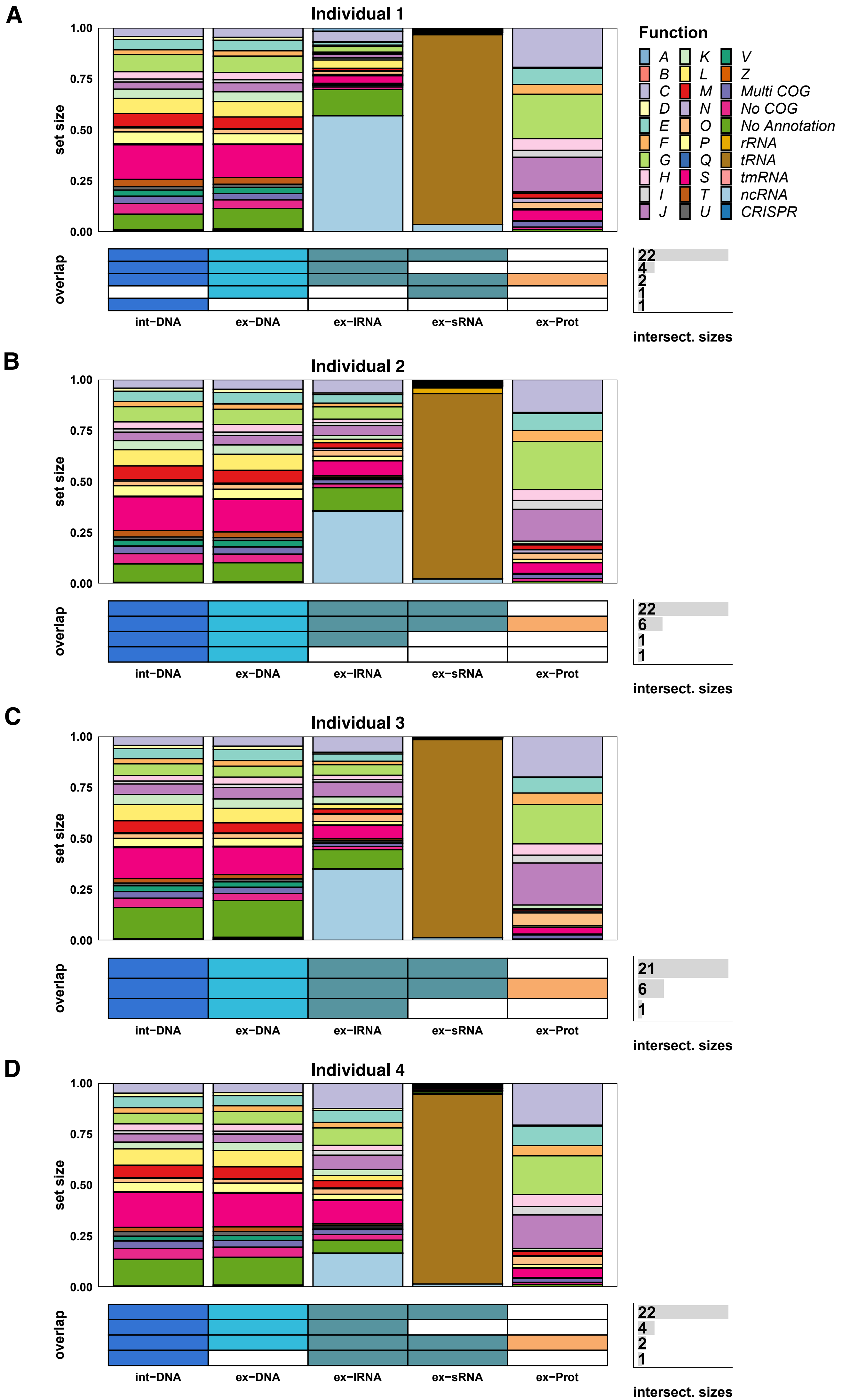
A
Individual 1
B
Individual 2
C
Individual 3
D
Individual 4
set size
1.00
0.75
0.50
0.25
0.00
overlap
int−DNA
ex−DNA
ex−lRNA
ex−sRNA
ex−Prot
intersect. sizes
22
4
2
1
1
22
6
1
1
21
6
1
22
4
2
1
Function
A
B
C
D
E
F
G
H
I
J
K
L
M
N
O
P
Q
S
T
U
V
Z
Multi COG
No COG
No Annotation
rRNA
tRNA
tmRNA
ncRNA
CRISPR

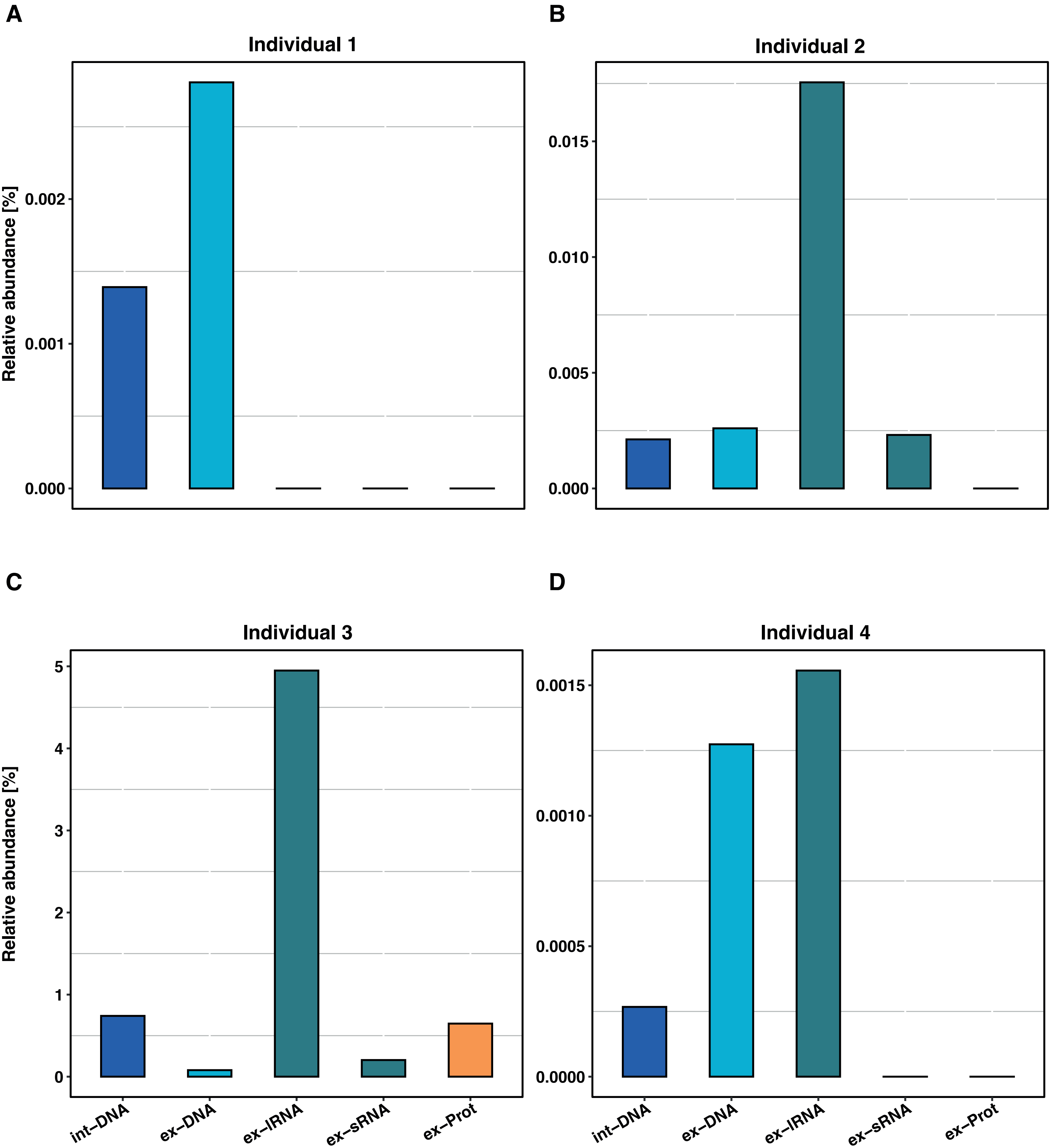

A
Individual 1
Relative abundance [%]
0.000
0.001
0.002
B
Individual 2
0.000
0.005
0.010
0.015
C
Individual 3
Relative abundance [%]
0
1
2
3
4
5
int–DNA
ex–DNA
ex–lRNA
ex–sRNA
ex–Prot
D
Individual 4
0.0000
0.0005
0.0010
0.0015
int–DNA
ex–DNA
ex–lRNA
ex–sRNA
ex–Prot

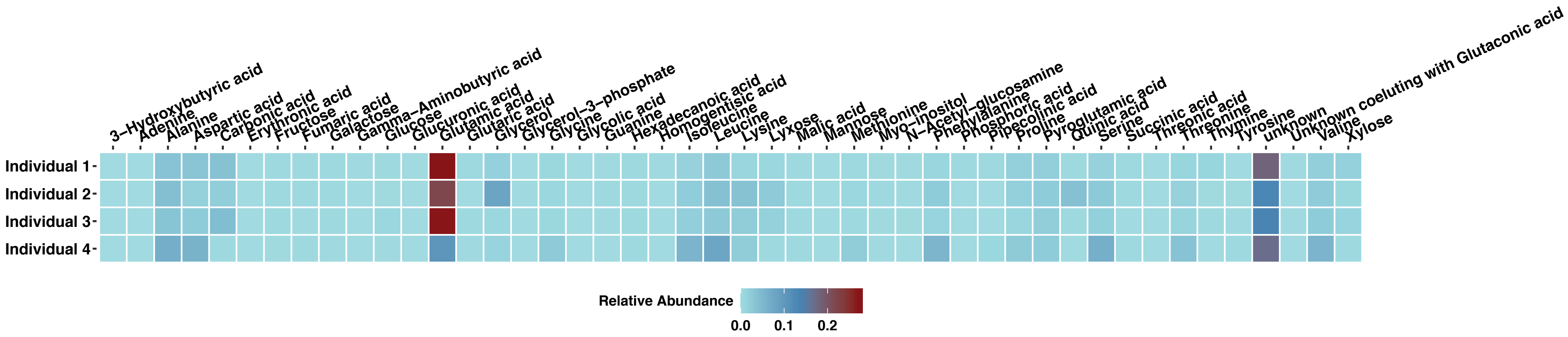

3-Hydroxybutyric acid
Adenine
Alanine
Aspartic acid
Carbonic acid
Erythronic acid
Fructose
Fumaric acid
Galactose
Gamma-Aminobutyric acid
Glucose
Glucuronic acid
Glutamic acid
Glutaric acid
Glycerol
Glycerol-3-phosphate
Glycine
Glycolic acid
Guanine
Hexadecanoic acid
Homogentisic acid
Isoleucine
Leucine
Lysine
Lyxose
Malic acid
Mannose
Methionine
Myo-inositol
N-Acetyl-glucosamine
Phenylalanine
Phosphoric acid
Pipecolinic acid
Proline
Pyroglutamic acid
Quinic acid
Serine
Succinic acid
Threonic acid
Threonine
Thymine
Tyrosine
unknown
Unknown coeluting with Glutaconic acid
Valine
Xylose
Individual 1
Individual 2
Individual 3
Individual 4
Relative Abundance
0.0
0.1
0.2
